# Silk Fibroin Surface Engineering Using Phase Separation Approaches for Enhanced Cell Adhesion and Proliferation

**DOI:** 10.1021/acsami.5c00874

**Published:** 2025-02-19

**Authors:** Karolína Kocourková, Markéta Kadlečková, Erik Wrzecionko, Filip Mikulka, Eliška Knechtová, Petronela Černá, Martin Humenik, Antonín Minařík

**Affiliations:** † Department of Physics and Materials Engineering, Faculty of Technology, Tomas Bata University in Zlín, Vavrečkova 5669, 760 01 Zlín, Czech Republic; ‡ Centre of Polymer Systems, Tomas Bata University in Zlín, Třída Tomáš Bati 5678, 760 01 Zlín, Czech Republic; § Department of Biomaterials, Faculty of Engineering Science, University Bayreuth, Prof.-Rüdiger-Bormann. Str. 1, 95447 Bayreuth, Germany

**Keywords:** cell interaction, phase separation, secondary structure, silk fibroin, surface texture, 3D printing

## Abstract

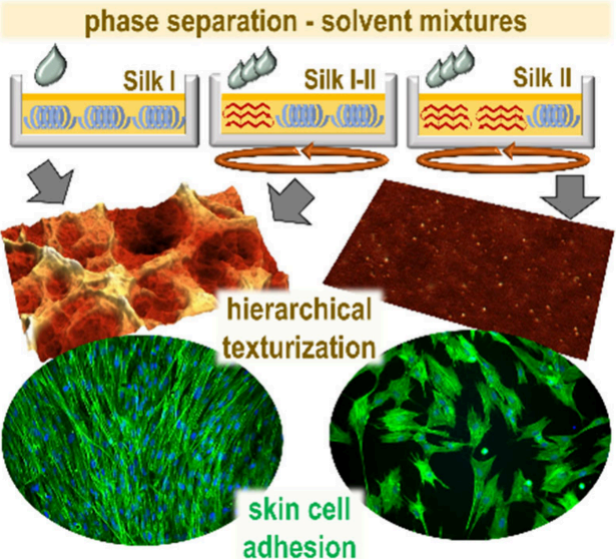

Due to excellent mechanical properties and biocompatibility, materials based on silk fibroin are increasingly included in advanced biomedical research and applications. However, their poor supporting properties for cell adhesion and proliferation represent limiting factors of the utilization. To eliminate this deficiency, we developed a series of phase-separation approaches allowing for tunable texturing of planar and 3D printed fibroin surfaces from nano to macro levels. The formation of surface structures presented is based on a combination of good and poor solvents, whereas no potentially problematic templates or additives, diminishing biocompatibility of the resulting material, are required. A critical factor in obtaining and scaling of the textures is control over the degree of transformation of fibroin secondary structures between prevalently amorphous Silk I and semicrystalline Silk II forms before and during surface treatment. Employing a set of optimized procedures, selectively or hierarchically structured fibroin surfaces can be prepared at the nano, micro, and macro level, which are characterized by long-term stability in physiological environments, allowing enhanced adhesion and proliferation of human keratinocytes as well as skin fibroblast cultivations.

## 1 Introduction

Biocompatibility combined with availability and variable processability made proteins and polysaccharides frequently employed sources of materials for current developments in biomedical applications.^1^ The limitations of biopolymer-based materials in terms of stability toward biodegradation, capability to interact with cells, or control of differentiation into tissue structures could be surpassed by a plethora of modification and processing approaches.^2,3^ Hence, through growing knowledge on relationships between the external treatment conditions and the internal molecular and supramolecular structures, silk fibroin, used for centuries in the garment industry, is nowadays becoming one of the most promising materials into the field of highly specific biotechnological applications.^4−7^ Silk fibroin (SF), which is derived from fibers of domesticated silkworms *Bombyx mori*,^8^ exhibit high biocompatibility comparable to that of collagen.^9^ Although SF does not contain motifs in the protein sequence supportive of cell attachment, this deficiency could be counteracted by chemical modifications with, for example, the cell-binding peptide RGD^10^ or also by surface texturization of SF-based culturing matrices.^11,12^ Currently, a range of material systems with specific properties that support cellular proliferation are being developed by utilizing, for example, the piezoelectric properties of materials. However, these systems often require more complex preparation and the combination of multiple types of materials to create intricate composite structures.^13^ The advantage of fibroin compared to other synthetic or natural polymers lies in controlling the secondary structure composition, which in turn allows tuning the material properties without the need for additives.^14−16^

Cells responded specifically and with high sensitivity to surface topography changes.^17−19^ Some of the earliest observations of cell–surface interactions were already obtained in the early 1900s, when Harrison showed an oriented migration of embryonic cells on spider silk webs.^20^ Generally, the formation of topographic patterns at nanometer scales leads to an increase in specific surface area, which promotes the adsorption of serum proteins, hence providing specific binding sites for cells, facilitating their interaction with the surface and promoting adhesion. In turn, surface patterns at microscales rather influence cell shape, migration, or differentiation behavior.^21,22^

The porosity and surface texture of a material play crucial roles in healing processes. In addition to the factors mentioned above, porous structures facilitate easier exchange of nutrients and metabolites.^23^ On the other hand, nonporous materials help maintaining a controlled microenvironment around the wound and better fulfill the barrier function.^24^ In some cases, the antifouling or antibacterial property of nonporous surfaces can also be beneficial.^25^ Porous materials can also exhibit lower mechanical strength.^26^

In the form of self-supporting films or coatings on various supports, SF is a suitable covering material for wound healing applications,^27^ revealing several advantages, such as easy sterilizability, transparency for better monitoring of regeneration progress, and suitable rate of degradation.^2,28^ However, missing cell adhesion supporting functionalities have limited the application of SF-based coatings.

The most common way to prepare macro, micro, or nano textures on SF films is soft lithography, employing a transfer of structures from polydimethylsiloxane (PDMS) templates.^29−35^ The multistep procedure involves preparations of one or more templates with a transferable pattern, which is an elaborate process, associated with higher costs and risk of sample contamination. Another approach used the formation of so-called breath figures (BF). The method is based on a phase separation of condensing and pore-forming micrometer-sized water droplets as a nonsolvent during evaporation of a good solvent from a softened polymer surface in a humid atmosphere. The rapid evaporation of the solvent causes system cooling, thereby promoting condensation and growth of the water droplets.^36,37^ The existing literature, however, evidences that the fibroin surface cannot be textured directly using the water-based BF method, as water readily interacts with the protein films acting as a softener as well.^38^ To prepare textured surfaces using phase separation processes, an admixture of poly(ethylene oxide) or paraffin spherical particles was applied.^39−41^ Furthermore, the preparation of porous fibroin structures was further carried out using photolithography,^12^ lyophilization,^42^ or elution of fibroin nanoparticles generated during SF-film autoclaving^43^ as well as electrospinning of nanofiber mats.^44^ The disadvantage of the methods developed so far is mainly the usage of additives or templates requiring subsequent removal, leading to obstacles with contaminants, diminished mechanical properties, or environmental stability of the prepared structures.

Herein we present a series of approaches based on phase separation effects in mixtures of good and poor solvents exerted on the surface of rotating or static SF films and printed grids containing different protein secondary structures ranging from Silk I (amorphous structure) to Silk II (semicrystalline) conformation. We show and elaborate on the interconnection between the degree of SF structural transformation and its texturing capability with relief widths and depths ranging from tens of nanometers to hundreds of micrometers. These differently textured surfaces were used to test the cell proliferation of human fibroblasts and keratinocytes with the aim of potential application in wound healing.

## 2 Materials and Methods

### 2.1 Preparation and Purification of Fibroin

Regenerated *Bombyx mori* fibroin was prepared in lyophilized form according to a standard protocol.^8^ The starting material from silk cocoons was deprived of sericin by boiling in 0.02 M Na_2_CO_3_. After being rinsed with water, the mass was dissolved in 9.3 M LiBr at 25% (w/v) concentration at elevated temperature (60 °C) for 4 h. After dissolution, the protein was transferred to deionized water using dialysis. After centrifugation and filtration, the extracted fibroin was lyophilized and subsequently dissolved in HFIP to a concentration of 2 or 3% (w/v).

### 2.2 Preparation of Fibroin Films

Fibroin films were prepared by casting 450 μL of the 2 wt % solution into polystyrene Petri dishes (TPP Techno Plastic Products AG) with a diameter of 3.4 cm. Drying was carried out in a flowing nitrogen atmosphere for 12 h to obtain the Silk I film. Subsequently, the films were placed either in a climate chamber with a defined relative humidity (60%) or with methanol vapor to get transient Silk I/II and Silk II film, respectively.

### 2.3 Texturization Using Sequenced Dosing and Substrate Rotation

For the purpose of surface texturing, a device built at the Department of Physics and Materials Engineering of the FT UTB was used.^45^ The surface of the fibroin film was modified by dispensing a mixture of good solvent HFIP, water, andnonsolvent DMSO in a ratio of 7:4:0.2 onto a rotating sample. The monitored process parameters were a sample rotation speed in the range 1500–2400 rpm, a dosing volume in the range 100–300 μL, a dosing period of 5–13 s, and several repetitive doses in the range 10–40. Each modification procedure was performed at least 10 times, using 3–5 samples per iteration. Due to slight structural variations at the edges of textured films, edge areas were excluded from evaluation. Over 80% of the central surface area, which exhibited consistency, was subsequently characterized.

### 2.4 Texturization on the Static Substrate

SF films in the Silk I condition were coated with a 450 μL solution HFIP/H_2_O/DMSO (volume ratio 7:4:*x*, where *x* was 0.1/0.2/0.4; for comparison see Figure S12). Samples were placed in a desiccator with a moderate flow of nitrogen until dry for 12 h.

In the second static modification approach, a layer of HFIP was deposited on the fibroin film to swell for 10 min and then placed in a chamber with a nitrogen and DMSO flow. The dry films were covered with a lid during drying to prevent unwanted surface wrinkling.

### 2.5 3D Printing of Grids

Fibroin grids were fabricated using electrohydrodynamic jetting (EHD) on a custom-built printer similar to that previously described for melt electrowriting.^46^ A solution of 3 wt % fibroin in HFIP was jetted onto a collector maintained at 50 °C (to accelerate the evaporation of HFIP) from a needle with a diameter of 160 μm. A polished silicon wafer with a thickness of 525 ± 15 μm was placed on the collector, which operated at a speed of 10 mm s^–1^. The applied voltage was set to 1.4 kV. The printhead was heated to 25 °C, with the relative humidity in the environment maintained at 30%. The EHD printing process is schematically illustrated in Figure S18B.

### 2.6 Texturization of Grids

After printing, the fibroin samples were treated under different conditions. Some grids were preserved as printed in the Silk I state, others were exposed to 60% relative humidity for 24 h, and a subset was treated with methanol vapor for 24 h. The changes from the Silk I to Silk II state were confirmed by FTIR (not shown). Subsequently, the grids were textured using a modification solution of HFIP/H_2_O/DMSO in a ratio of 7:4:0.2. A 10 μL aliquot of the solution, precooled to 4 °C, was applied to the grids without sample rotation, followed immediately by drying with an air stream.

### 2.7 FTIR Analysis

The analysis of the secondary conformation of fibroin was performed using a Nicolet iS5 FTIR spectrometer by the ATR technique with Ge crystal. 32 scans were recorded at a resolution of 4 cm^–1^ in the range 400–4000 cm^–1^. For each condition of silk fibroin film, measurements were performed on at least five samples. The FSD procedure was performed as described in Humenik et al.^47^

### 2.8 Profilometry

The topographic changes of the surface were characterized with a Contour GT-K optical profilometer (Bruker) using white light and a 20× magnification lens. Cross sections of images from optical profilometry were obtained by a Dektak XT mechanical profilometer (Bruker) with a diamond tip with a radius of curvature of 2.5 μm and a pressure of 5 mg.

### 2.9 Atomic Force Microscopy

Detailed topographic changes were characterized using a Dimension ICON (Bruker) scanning probe microscope. Measurements were performed at a scan rate of 0.7 Hz with a resolution of 512 × 512 pixels in tapping mode at room temperature in air. A probe with a resonant frequency of 70 kHz and a stiffness constant of 0.4 N/m (ScanAsyst-Air, Bruker) was used.

### 2.10 Scanning Electron Microscopy

Printed grids were observed by scanning electron microscopy (SEM) with Phenom XL G2 (Thermo Fisher Scientific). Prior to SEM, the samples were sputter coated with a mixture of gold and palladium in a Quorum SCSC7620 Mini sputter coater (Quorum Technologies) (argon gas, 15 s sputter time) to increase sample stability for analysis. Samples were analyzed at an acceleration voltage of 15 kV with backscattered and secondary electron detectors (50% mix) with Image or Mapping mode.

### 2.11 Goniometry

The water contact angle (θ) was characterized on the silk surface using a DSA30 Drop Shape Analyzer and ADVANCE 1.7 software (KRÜSS) for automatic image analysis. Measurements were performed at room temperature (298 ± 1 K). A drop with a volume of 3 μL was deposited on the measured surface. Ultrapure water with a resistance of 18.2 MΩ cm was used for the measurement. All measurements were repeated 9 times.

### 2.12 Cell Adhesion and Proliferation

Proliferation of keratinocytes (HaCaT) and human fibroblasts (BJ) was investigated on selected textured surfaces. Samples were sterilized by UV-irradiation for 1 h prior to in vitro testing. DMEM medium containing 10% calf serum, 1% glutamine, and 0.1% gentamicin was used to culture osteoblasts. Cells were plated on sterile textured surfaces at an initial concentration of 5000 cells/cm^2^ (22500 cells/mL). Cell proliferation was assessed after 3, 5, and 12 days by the alamarBlue assay. Cell elongation was expressed using an aspect ratio parameter, which is the ratio between the major and minor axes of the cell, calculated by using ImageJ software. The cell density (number of cells per mm^2^) was also analyzed using ImageJ software. An example of nuclei and cell shape outlining is shown in Figure S16 in the Supporting Information.

### 2.13 Statistical Analysis

The images, profiles, and surface roughness parameters were processed using the Gwyddion – Free SPM data analysis software, version 2.55 (D. Nečas, P. Klapetek, Czech Metrology Institute, Czech Republic). To cover the complexity of the surfaces, we calculated three roughness parameters Sq, Sa, and Sz, using Gwyddion on three samples of each surface type; the distribution of pit diameters and mean value were obtained based on an analysis of four samples. All surface roughness results, as well as pit diameters, were obtained from AFM images or optical profilometer images and are shown as mean value ± standard error of the mean. The surface pit quantity was analyzed by the ImageJ software, version 1.5 (W. Rasband, National Institutes of Health, United States). The statistical significance and *p* values were analyzed using one-way ANOVA followed by a posthoc Tukey test.

## 3 Results and Discussion

### 3.1 Fabrication of Macro-, Micro-, and Nanostructured Silk Substrates

**Figure 1:**
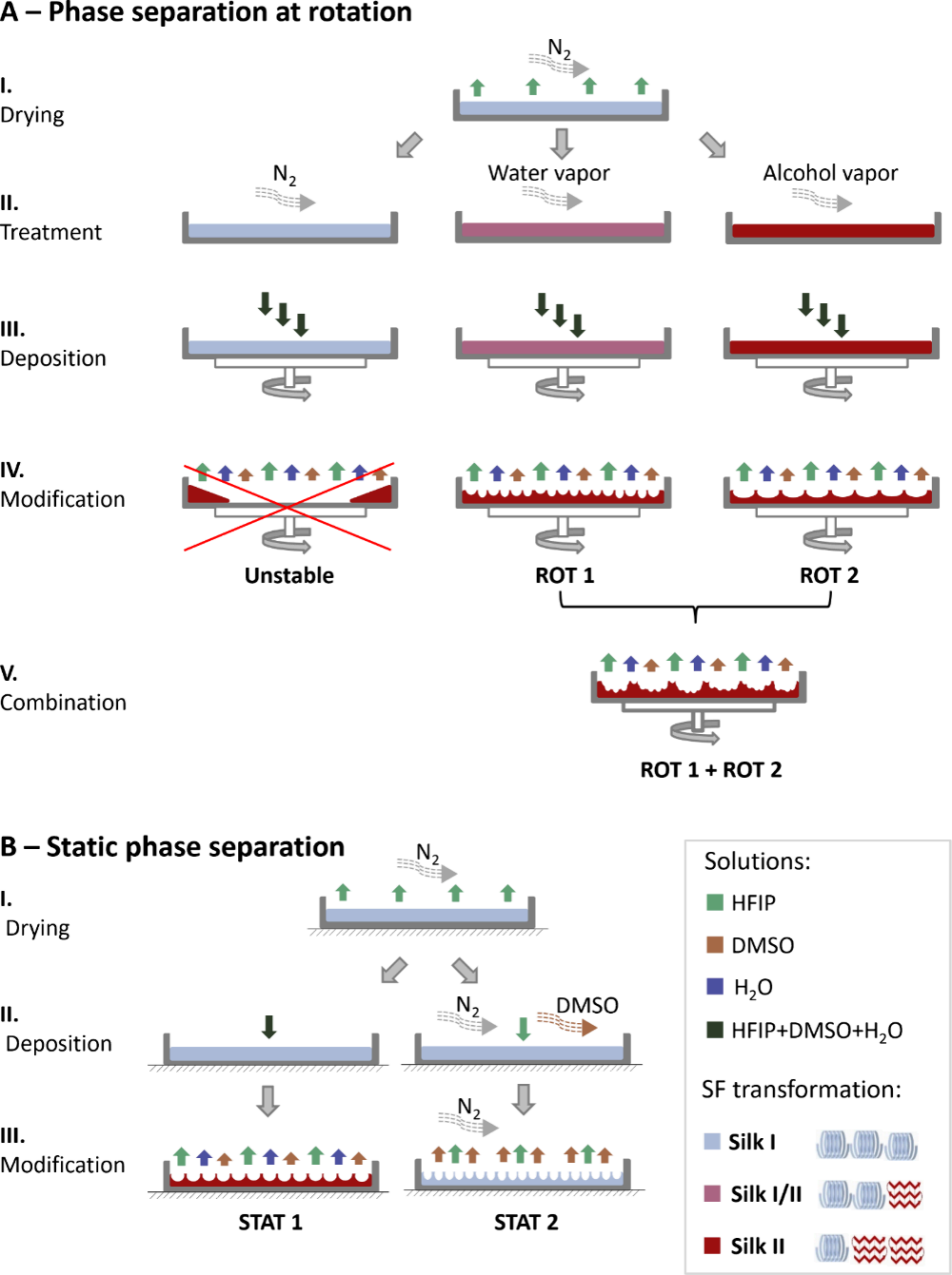
Schematic representation of processes to prepare textured SF films using (A) rotating substrate and sequential solvent dosage and (B) one dose of a solution mixture (left) or static substrate under DMSO vapor (right). I–V indicate individual steps in A and B, respectively. Hexafluoroisopropanol (HFIP) was used as a good solvent, dimethyl sulfoxide (DMSO) as a nonsolvent, and water as a plasticizer. Silk I represents an SF film state (light blue) with amorphous random coil/helical protein conformations obtained upon casting from HFIP solution, Silk I/II is a transient state (pink) obtained upon Silk I treatment with water vapor (atmosphere at 60% relative humidity (RH)) and Silk II represents semicrystalline beta-sheet rich films (dark red) transformed from Silk I upon treatment with alcohol vapors or at the end of some texturing procedures.

Several differently modified phase separation approaches were used to texturize SF surfaces in this study (Figure 1), employing either a rotating substrate and a sequential (multiple) dropping of mixed solvent/nonsolvent mixtures (A) or static films under the influence of DMSO vapor or single dose of solvent/nonsolvent mixtures (B). The secondary structure of SF was found to condition both the solubility and stability of the textured protein layer and the possibility of forming different nano-, micro-, or macrostructures and combination thereof.

### 3.2 Influence of Phase Separation on Surface Texturing at Substrate Rotation

The methodology for preparing textured surfaces by a sequential dispensing of mixed solvents is based on our study, where a solution containing good and poor solvents was dispensed in defined volumes onto a rotating polystyrene substrate.^45^ The good solvent swelled the synthetic polymeric material, whereas the nonsolvent phase-separated on the surface and formed droplets served as a pore templating agent. The possibility of applying this principle to biopolymer-based materials was demonstrated on hyaluronan coatings. In this case, we found the role of water as a plasticizer which significantly affected the mobility of polymer chains in the surface layer.^48^ However, both materials have several limitations that hinder direct applications in tissue engineering, e.g., nondegradability of the PS or water solubility of hyaluronan. Therefore, the phase separation process was adapted for fibroin, an FDA-approved protein system with sufficient biocompatibility, stability in aqueous environments, and biodegradability.^7^ In the presented work, hexafluoroisopropanol (HFIP) was chosen as a good solvent and dimethyl sulfoxide (DMSO) as a nonsolvent. In addition, the modification mixture contained water as a plasticizer, preventing film cracking due to mechanical stress during modification solvent evaporation (Figure S1).

**Figure 2:**
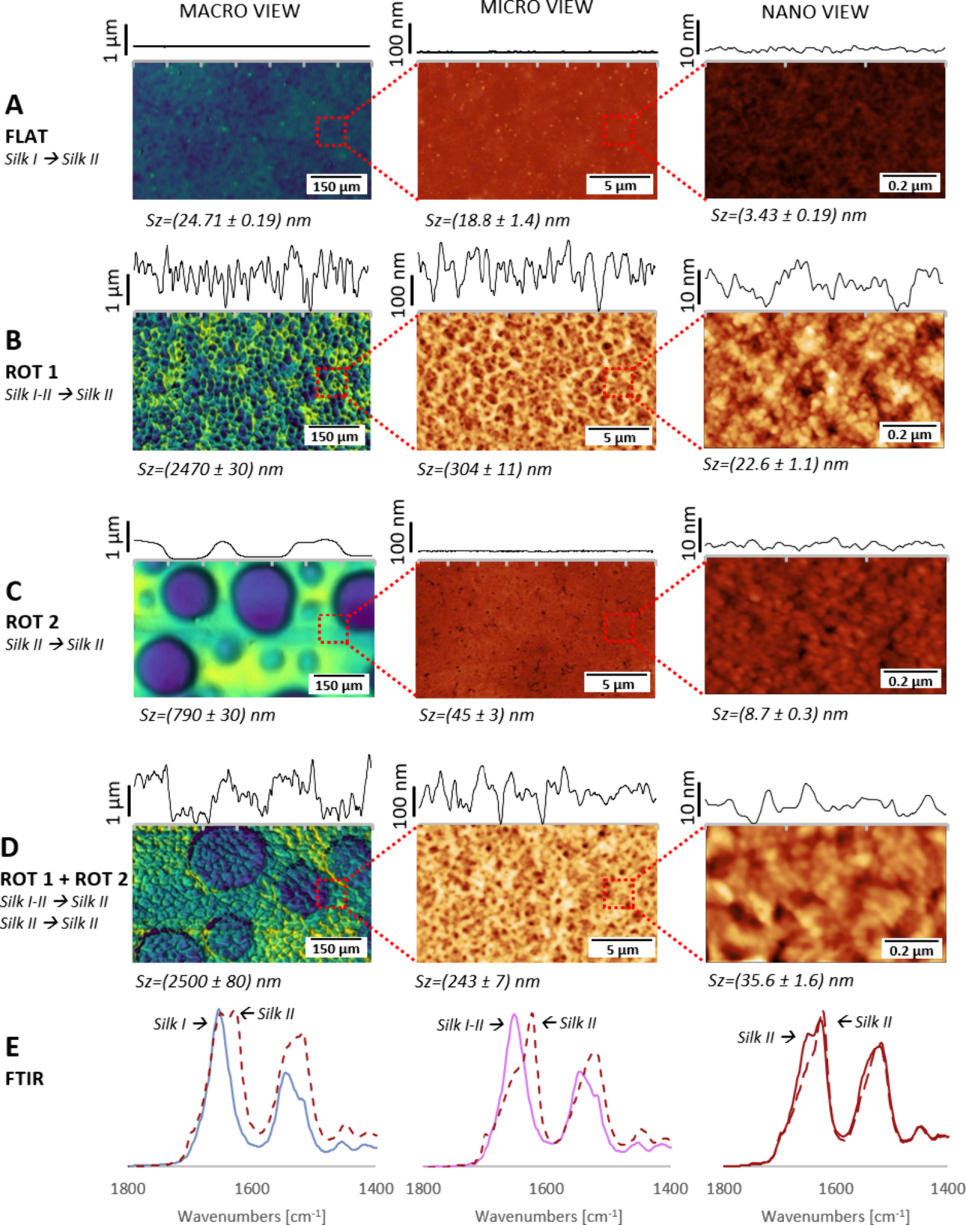
Different types of textured SF surfaces prepared using a sequenced dosing of solvent mixtures at substrate rotation. (A) FLAT - smooth, methanol treated Silk II film before surface modification. (B) ROT 1 - nano to macro pit pattern prepared on the transient Silk I/II film (Figure S2B) using a mixture of HFIP, H_2_O, and DMSO (7:4:0.2 volume ratio) and dosing 40 × 100 μL at 1500 rpm, delay between the doses 10 s. (C) ROT 2 - macro pit pattern prepared on Silk II films (Figure S2C), the same mixture as in (B) and dosing 10 × 200 μL at 2400 rpm, delay between the doses 5 s. (D) ROT 1 + ROT 2 - hierarchically textured surface prepared by a combination of the treatments in (B), which also caused transformation of Silk I/II into Silk II films, and (C) afterward. Left panels were obtained with an optical profilometry and middle as well as right panels with AFM. (E) FTIR spectra of SF films in different preparation steps. Left panel: blue solid line shows the initial film in Silk I state and red dashed line after treatment with methanol vapor (structure in (A)). Middle panel: pink solid line shows the transient Silk I/II state obtained from Silk I (blue) after 60% RH treatment, and the red dashed spectrum shows the change to Silk II state after the solvent dosing (structure in (B)). Right panel: red solid line shows the Silk II state after methanol vapor treatment, and red dashed line represents the spectra after the solvent dosing on the Silk II film (structure in (C)).

In contrast to the polystyrene material, fibroin films could be textured at different initial stages, differing in the content of protein secondary structures. The films were initially prepared using drop-casting of a 2% (w/v) fibroin solution in HFIP into Petri dishes. Importantly, the films were dried in a nitrogen flow to exclude moisture (Figure 1A-I), thereby enabling achieving net Silk I conformation as verified by FTIR (Figure 2E, blue spectrum, solid line). Without the N_2_ environmental control, the film conformational state varied from batch to batch due to changing air humidity (not shown), hence hindering reproducibility of the texturing processes. Moreover, rapid evaporation of the volatile HFIP resulted in undesirable wrinkling on the films induced by temperature and concentration gradients.^49−51^ Nevertheless, a simple covering of the films with a cap was sufficient to eliminate this phenomenon. However, employing sequenced dispensing of solvent mixtures (Figure 1A-II–IV, left) resulted in the material washing off. Hence, only static approaches could be applied to the Silk I films, as discussed later (Figure 1B and Section 3.3). To increase the solvent stability of the SF films, protein secondary structures in the N_2_-dried films were transformed by methanol vapors, as shown by FTIR analyses (Figure 2E, Figure S3). The resulting shifts of the maxima in characteristic Amid I and II regions were consistent with published observations.^52,53^

The films in the amorphous Silk I state have shown typical maxima for prevalently random coil/α-helical structures at 1654 and 1546 cm^–1^ in Amid I and II region, respectively (Figure 2E, left, blue spectrum, Figure S3A, Table S1). The methanol treatment caused a transition to the Silk II conformation with a typical β-sheet rich content (maxima at 1627 cm^–1^ (Amid I) and 1518 cm^–1^ (Amid II), Figure 2E, right, red solid line, Figure S3C, Table S1). Different times of methanol vapor exposure (24/48/72 h) on the conformational change were tested (Figure S3D–F, Figure S4, and Table S1), showing 48 h to be a sufficient transformation time. Apart from the methanol treatment, causing β-sheet rich Silk II state of the films, we also tested the influence of 60% RH for 48 h, inducing transient Silk I/II state (Figure 2E, middle, pink spectra, Figure S3B) showing slightly shifted maxima to 1650 and 1544 cm^–1^ in comparison to Silk I state (blue) due to less α-helical content and slight enrichment with β-sheet structures (Table S1). Further conformational changes were observed during surface modifications using different solvent mixtures (Figure 2E, red dashed lines). The changes in the Silk I film (blue spectrum) upon treatment under static conditions are described in Section 3.3.1. In the transient Silk I/II film (pink spectrum) a complete transformation to Silk II occurred after the dosing of HFIP/DMSO/H_2_O solvent mixture, as well as in the case of the film in the Silk II state (red solid line) showing further decrease in random coil and helical conformations (1651 cm^–1^) and an increase in the content of β-sheet structures (1624 cm^–1^).

The transformed, i.e., structurally and environmentally stabilized, films enabled rotational dosing of the solvent mixtures (Figure 1A-I,II, middle and right) and formation of hierarchical surface porous textures, whose architecture was determined by the initial conformational state. Hierarchical topographies have been observed at several levels, which have been defined as follows: nano referring to elevations <1 μm, micro 1–30 μm, and macro >30 μm. The surface of the initial, methanol converted Silk II films is shown in Figure 2A - FLAT. At the microscopic scale, the film appeared smooth, but in the detailed AFM images, the rougher nanostructure formed in comparison to Silk I film (Table S2, Figure S2A vs Figure S2C, upper panel) similarly to the treatment with 60% RH (Table S2, Figure S2B, upper panel).^54^ The change in nanotopography was accompanied by changes in adhesiveness, although the rougher transformed films revealed lower “stickiness” in comparison to the initial Silk I film most probably due to compaction of the loose protein chains in random coil structures into compact beta-sheet clusters (compare Figure S2A vs Figure S2B,C, lower panels).

#### 3.2.1 Formation of Combined Nano to Macro Texture

When sequenced dosing of a mixture containing HFIP, H_2_O and DMSO was applied to the films in the Silk I/II transition state (Figure 1A middle), a hierarchical pit pattern texture labeled as ROT 1 was formed on the surface at three levels (Figure 2B, Table S3). Level 1, shown by optical profilometry, was composed of micropits with a maximum elevation of ∼2 μm. A nanopit texture with 10× lower elevation was observed in a more detailed AFM image, and level 3 showed a more pronounced nanotexture than the smooth nontreated films, as can be seen in Figure 2A and Table S3 with roughness parameters. This type of hierarchical texture remained stable in water or methanol after 24 h (Figure S5I-A–C , roughness parameters in Table S4), which was important for washing out any residual solvents with phosphate buffered saline (PBS) and subsequent sterilization with alcohol before cell proliferation tests. In culture medium at 37 °C, the structure began to swell after 7 days of exposure, which is reflected by an increase in roughness parameters and a subsequent loss of texture (Figure S6I-A–C, Table S5).

#### 3.2.2 Formation of Macro Texture

Using the films in the Silk II conformation (Figure 1A, right) and the sequential solvent mixture dropping at rotation formed a significantly different texture consisting of circular depressions with diameters of (135 ± 2) μm (Figure S11) labeled as ROT 2 (Figure 2C). Detailed texture of spaces in and between the depressions resembled, however, the smooth surface of nontreated Silk II film but with approximately doubled roughness parameters (Table S3). We observed that the resulting textures were determined by the amounts of DMSO and H_2_O in the modification mixtures, whereas the water apparently functioned as a surface plasticizer (Figures S1 and S7). Similarly, further process parameters had a significant influence on the emerging pits, resulting in an optimal combination of substrate rotational speed (2400 rpm), number of doses (10), and delay between the doses (5 s) to achieve a homogeneous texture (Figure S8). Pretreatment time of the films with methanol (Figure S4) also exerted slight differences in the resulting surface texture density and an increase in surface roughness parameters (Figure S9, Table S6). Unlike the nano/micro pit pattern, the macrotexture disappeared either partially after incubation in methanol or fully in water (Figure S5-IIB,C, Table S4) due to film swelling. This phenomenon might by related to the fact that transformed SF films treated with methanol have a greater tendency to absorb water compared to transformed films in which changes in secondary conformation were achieved by water annealing.^55^ For this reason, the macro texture was not suitable for cell adhesion and proliferation assays (Section 3.4).

The significant difference in the emerging textures depending on the SF conformational state in the films (Silk I/II vs II) suggested different mechanisms of the structure formation. We assumed that in the case starting with the partially transformed Silk I/II film, the texture is formed due to competitive processes with HFIP partially dissolving the amorphous fibroin part on the one site and the concomitant pore-forming function of DMSO and transformation to the more stable Silk II with water on the other site. In contrast, for the fully transformed Silk II film, the surface is most probably just swollen by HFIP, plasticized by water, and deformed by the action of coagulated DMSO droplets. We proved this hypothesis using a fluorescein isothiocyanate labeled dextran (FID) (Figure S10A) dissolved in DMSO part of the modification solvent mixture (Figure 2C). Interestingly, the dye was found to mainly concentrate in the resulting depressions (Figure S10C).

#### 3.2.3 Combined Hierarchical Texture

Using the above-mentioned solvent mixture HFIP, H_2_O, and DMSO (7:4:0.2 ratio) in sequential dosing on the rotating film in Silk I/II state led first to a ROT 1 pattern (Figure 2B), and this textured film (transferred to Silk II by water in the solvent mixture) was subsequently subjected to rotational dispensing of the same mixture, forming the second macro texture ROT 2 (Figure 2D). The formation of this secondary macro texture while retaining the primary ROT 1 pattern again confirmed our assumption of the pore-forming function of DMSO without the Silk I layer washing off.

### 3.3 Static Phase Separation

#### 3.3.1 Good and Poor Solvent in One Dose

**Figure 3:**
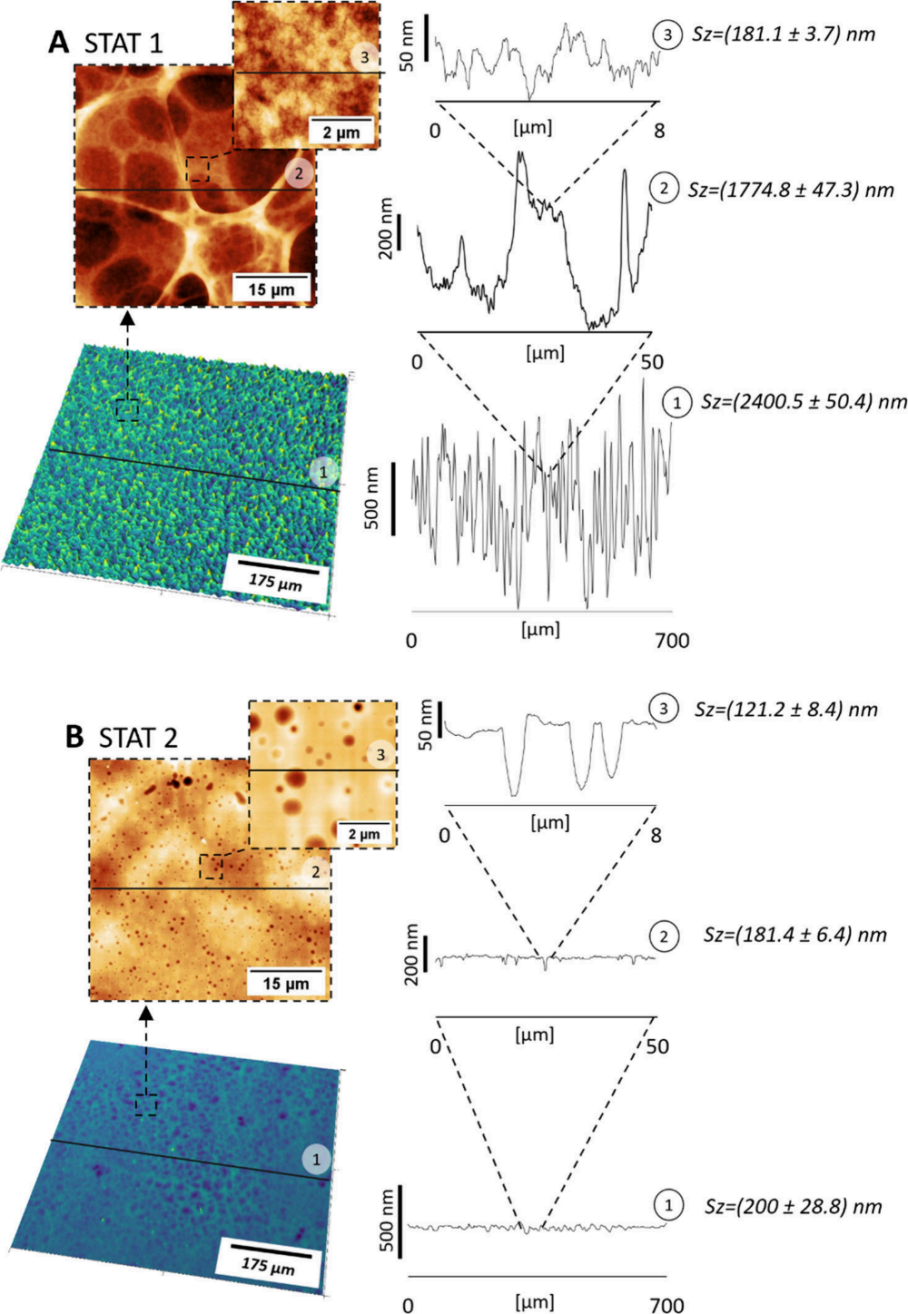
Porous SF films prepared on static substrates either using solution mixture HFIP/H_2_O/DMSO 7:4:0.2 in one dose (450 μL) in (A) or separately adding HFIP to swell the film and DMSO vapor to induce breath figures effect in (B). The lower panel shows profilometry images (green/blue), and the two upper panels (brownish) show AFM images with corresponding cross sections.

Due to the washing off in the case of the Silk I film (Figure 1A, left) during the rotational dispensing, static modification approaches were set up (Figure 1B). The first type, STAT 1, was a single dosing of the mixture containing good solvent (HFIP), nonsolvent (DMSO), and plasticizer (H_2_O). The result is shown in Figure 3A, with the porous texture formed at multiple levels (see roughness parameters in Table S7). The character of the texture can be modified by the concentration of the nonsolvent, where the ability to form this specific topography was diminished with the increasing proportion of DMSO also influencing the surface roughness (Figure S12A–C, Table S8). This type of modification resulted not only in topography changes but also in a concomitant conformational transition to a water-resistant Silk II structure (FTIR spectra, Figure 2E, transition blue to red dashed line) similar to the nano/micro pattern prepared at rotation. Hence, the prepared surface exhibited texture stability in a cell culture medium up to day 7 (Figure S13 and Table S5).

#### 3.3.2 Breath Figures-Like Approach with DMSO Vapor

Using the static Silk I film, we developed a modified method based on the breath figures approach. The surface of the fibroin film was first swollen with HFIP for 10 min and placed in a chamber with flowing nitrogen enriched in DMSO vapor, which took over a similar function as condensing water droplets in the conventional BF process.^36^ However, the fibroin in SILK I state is water-soluble, which hinders the utilization of water-based breath figures texturing.^38^ Condensation of DMSO droplets on the cooling fibroin surface due to HFIP evaporation resulted in the formation of isolated circular depressions on the fibroin film labeled STAT 2 (Figure 3B). DMSO evaporates very slowly at room temperature, so the evaporation rate was increased by heating to 45 °C. Importantly, nitrogen was bubbled through the solvent, to increase the evaporation surface area as well as an inert gas carrier to sweep away the DMSO vapors from the liquid surface. This maintained a DMSO concentration gradient that favored continuous evaporation due to reduced vapor pressure (Figure S14).

**Figure 4:**
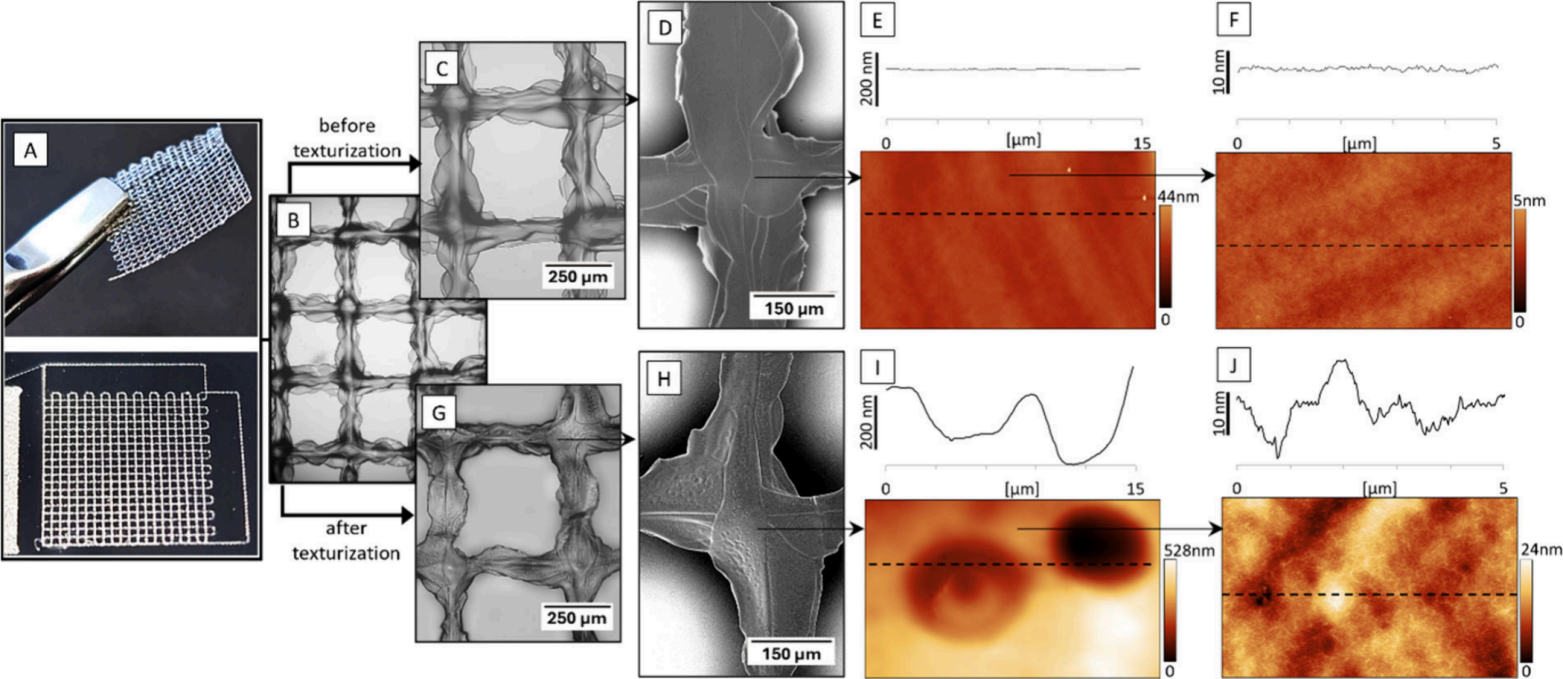
Texturization of EHD-printed fibroin grid (dimensions of 1 × 1 cm): (A) macroscopic view of the grid; (B) detailed view of the grid after posttreatment in 60% RH; (C–F) individual surface levels of the nontextured grid; (G–J) individual surface levels of the textured grid. The morphologies were visualized using an optical microscope (B, C, and G), SEM (D and H), and AFM with profile sections (E, F, I, and J).

Further, we demonstrate that the above-described phase separation processes are potentially applicable to fibers of 3D scaffolds. Using a 3D printing setup based on either electrohydrodynamic jetting (EHD) (Figure S18B) or microextrusion printing (Figure S18 A), solid grid-shaped objects were fabricated from fibroin solution in HFIP, as shown in Figure 4A–F and Figure S20, respectively. To achieve the control over the silk secondary structures, as described for the films above, the EHD grids were treated with either increased humidity (60%) or methanol vapors prior to surface modification. The texturization of the grids was performed using the same solvent mixture as that for the films. When this mixture was applied to the as-printed grids (SILK I state), the grids partially disintegrated, as shown in Figure S19A,B. In contrast, the methanol-treated grids (SILK II state) remained stable during the texturization process (Figure S19C,D), with only slight changes in surface texture noted at the nanoscale, as shown in Figure S19E,F. The grids exposed to 60% humidity (in the transitional state of SILK I-II) exhibited slight wrinkling (Figure 4D), primarily caused by the overlapping of individual printed layers and solvent evaporation during drying, but no pronounced texture was observed in the AFM images (Figure 4E,F). However, the samples exhibited changes in surface topography at several levels (Figure 4G–J) upon application of the solvent mixture. The SEM image (Figure 4H) reveals surface wrinkling with larger depressions (detail in Figure 4I) surrounded by a homogeneous pit-like texture (Figure 4J). The texturization was performed on the static substrate by a single-step application of the solvent mixture followed by immediate drying, since the microfibers were too susceptive to repetitive dropping under the rotational conditions. A comparison of rotational and static modifications was subsequently carried out on macroscopic grids obtained via microextrusion printing (Figure S20). In both cases, a specific surface texture was generated on the walls of the printed struts (Figure S21). As there is no processing technique known, which enables one to print solid 3D scaffolds from fibroin melts, the utilization of the phase separation on pure 3D fibroin scaffolds is currently limited. Nevertheless, the potential of the texturing methods could be enrolled in future on some synthetic biocompatible polymers such as polycaprolactone (PCL) or poly(vinyl alcohol) (PVA), commonly utilized in additive manufacturing and tissue engineering.^56^

### 3.4 Cell Proliferation on Structured Silk Substrates

**Figure 5:**
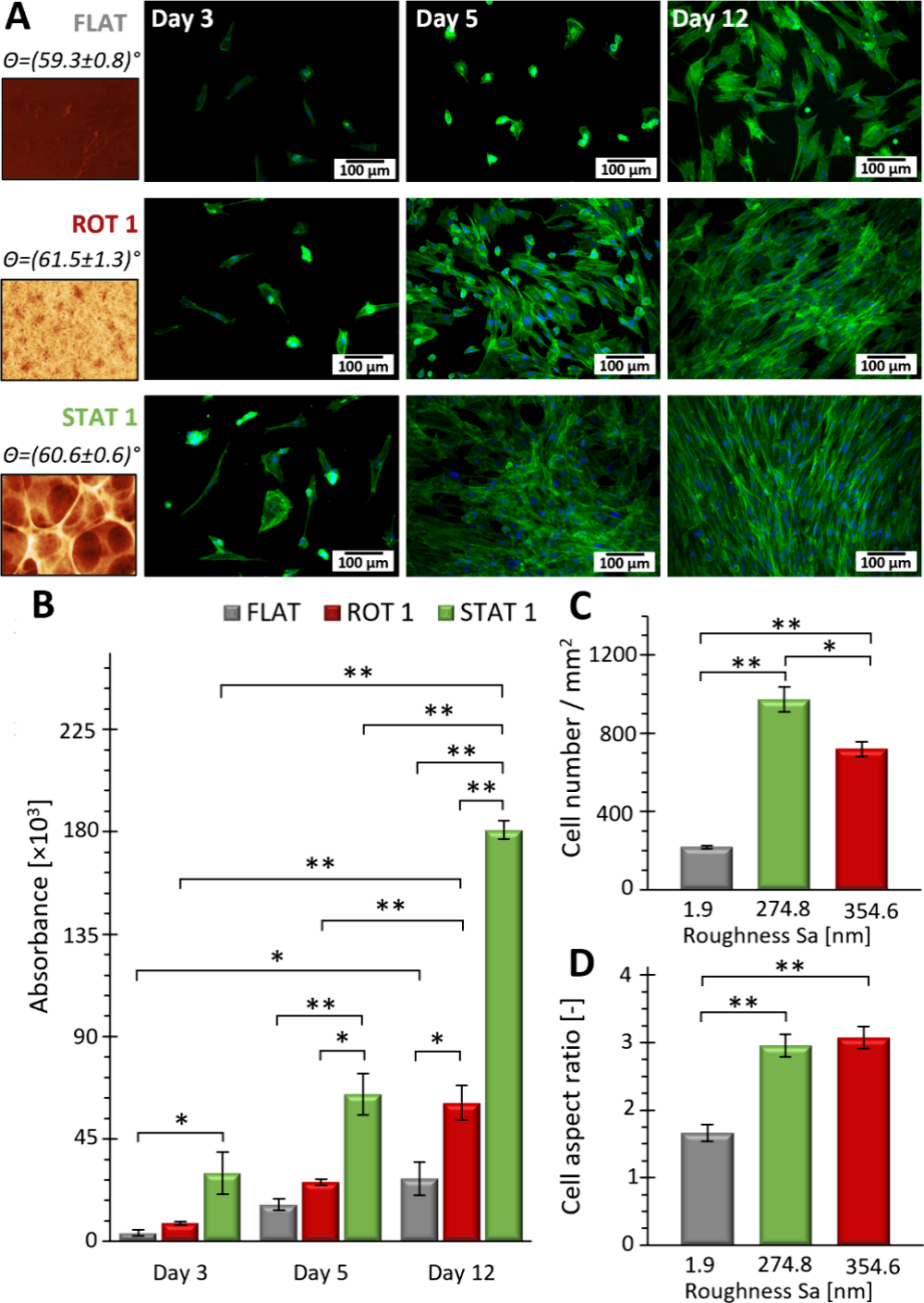
Human fibroblast proliferation on the SF films. (A) The cells were incubated on films indicated as FLAT = smooth SF surface (Figure 2A), ROT 1 = surface with nano/micro pit-pattern prepared by sequenced rotation dosing (Figure 2B), and STAT 1 = surface prepared by static modification (Figure 3A). Cell morphology was monitored using fluorescence microscopy after Nuclei (DAPI (blue)) and Actin (Phalloidin (green)) staining at the indicated day. (B) Corresponding proliferation assays on the films in (A) at days 3, 5, and 12. (C) Cell density (cells/mm^2^) after 12 days of proliferation and (D) cell aspect ratio after 5 days of proliferation in relation to the texture type, expressed as an average roughness (Sa) calculated over large area of 0.7 × 0.7 mm (see Tables S3 and S7). Statistically significant differences were determined using one-way ANOVA followed by a posthoc Tukey test; *p<0.05, **p<0.01.

Despite the good biocompatibility of fibroin-based materials, cell adhesion to unstructured SF substrates is typically not sufficient. However, the cell–substrate interaction could be supported by specific surface textures.^19^ Surface topography with elevations on the order of tens to hundreds of nanometers increased the contact area between the substrate and cell receptors, which could facilitate the cell attachment.^17^ In this work, the proliferation of a keratinocyte (HaCaT cell line) and human fibroblasts (BJ cell line) was studied on the textured surfaces. Both cell lines served as important models in skin healing studies.^57−59^ The smooth SF films were used as a reference (Figure 2A and Figure 5-FLAT). The cells were cultured on the nano/micro pit-patterned films prepared by the sequenced rotational dispensing (Figure 2B and Figure 5-ROT 1) and on the statically modified porous films (Figure 3A, Figure 5-STAT 1). However, macro pit-patterned films (Figure 2C-ROT 2) were not suitable for the assessment of texture-cell influence because of the texture instability in aqueous media (Figure S5II-C). Hence, the cell proliferation on this type of surface was similarly low as on the nontextured FLAT surface (Figure S15).

Figure 5 shows the proliferation of the human skin fibroblast over 12 days. Both textures, ROT 1 and STAT1, positively affected cell proliferation in comparison to the smooth films, as evidenced from the alamarBlue assay, showing increasing metabolic activity (Figure 5B), as well as from significantly higher cell surface densities (Figure 5C and FigureS16A–C) after 12 days of proliferation. The surface textures ROT1 and STAT1 also supported the characteristic elongation of the skin fibroblasts, expressed by aspect ratio (Figure 5D and FigureS16D–F), which nearly doubled in comparison to that on the smooth films after 5 days of proliferation. Even more pronounced cell elongation and alignments were apparent after 12 days; however, reliable quantification was not possible at this stage due to cell confluence.

Micro- and nanostructured materials exhibit high specific surface area which could decisively influence wetting properties.^60^ Hence, water contact angles on the SF films were measured to assess the impact of possible surface wettability changes on the cell behavior due to the multilevel texturing. The contact angle of the smooth fibroin treated with methanol was around 59°, which corresponds to the literature values. Methanol treatment leads to the formation of β-sheet structures with hydrophilic NH and CO groups enclosed inside during chain folding, resulting in a decrease in surface energy.^61^ Surprisingly, by generating the surface textures, the contact angle increased only slightly above 60° for both the STAT and ROT types. Hence, we assume no significant contribution of the wettability changes to observed cell adhesion. The prepared textures revealed contact angles in the generally accepted optimal range of 60–70°, which allows for the adsorption of amphiphilic biomacromolecules secreted by the cell to the surface facilitating cell adhesion.^62^

Although the textures ROT1 and STAT1 revealed similar physical properties (wetting and roughness paraments), the hierarchically porous surface STAT 1 provided the best results of dense cell coverage with typical alignments,^63,64^ even showing lower average roughness (Sa parameter) compared to the rotationally textured substrate (Figure 5C, Table S7 vs S3 at 0.7 × 0.7 mm area). Apparently, the form of the texture played an important role as well in the supporting cell proliferation, as the type of the hierarchical pore-like texture (STAT 1) appeared more advantageous in comparison to the pit type topography (ROT 1). Hence the texture-to-cell proliferation relationship cannot be expressed by or related to simple numbers such as wettability and/or average roughness, as they do not encompass the shape of the surface structure and complex hierarchies thereof.

Additionally, we also tested HaCaT cell line (keratinocytes), which is crucial for wound healing applications.^57^ Interestingly, no relationship has been seen between cell growth and the texture type, since the cells were capable of comparable proliferation on the FLAT, ROT 1, and STAT 1 surface, forming confluent multilayers of similar metabolic activity (Figure S17). In future, tests of a layered coculture of the keratinocytes (Figure S17) on the skin fibroblast (Figure 5) will be required to evaluate in detail the STAT 1 and ROT 1 substrates for supporting the development of coculture in a dermis type of tissue, which is prerequisite for tissue engineering application.^65^

## 4 Conclusion

Silk proteins are suitable biocompatible systems for a range of biological and medical applications. A major drawback of these materials is the weak cellular adhesion on their surface. This problem can be overcome by surface modification approaches. In this work, several phase separation-based methods have been developed leading to the preparation of macro-, micro-, and nanostructured fibroin surfaces. It has been shown that the formation of such structures is conditioned by the degree of the protein secondary structure transformation in the films, as well as the processing conditions during these modifications. Here the solvent HFIP, nonsolvent DMSO, and plasticizer H_2_O in different ratios are carefully chosen and added either sequentially on a rotating sample or in one portion on the static substrate. Whereas HFIP is important to swell the surface, DMSO causes pit-pattern formation. In the case of the Silk I-II state, erosion caused by the partial dissolution of the amorphous part of the fibroin is also involved in the formation of the nano/micro dimple texture. The transformed and environmentally stable Silk II structures are resistant to quick erosion by a small amount of HFIP, but DMSO droplets sink in the swollen HFIP substrate layer and cause macro pits. Silk I substrates are unstable toward sequential dosing; however, one-dose application of HFIP/DMSO/H_2_O caused formation of hierarchical structure nano to micro porous texture. We also showed that the breath figures approach could be adapted to the SF film in the Silk I state using DMSO instead of water vapor. Nano/micro and hierarchically porous textures were stable in cell culture environments for several days, which was fully sufficient to increase adhesion and proliferation of keratinocytes and skin fibroblasts that are central to wound healing applications. Hence, employing quite simple processing involving a small subset of solvents in combination with droplet dosing and a static or rotating silk fibroin film and control over the protein secondary structures, a wide range of textures combining nano, micro, and macro porous structures is possible. This approach expands the variability in textured and porous surfaces typically achievable with biocompatible materials by electrospinning. Furthermore, textured films and 3D printed grids are mechanically more robust than electrospun matrices, which can be advantageous for applications requiring higher mechanical stability and integrity.^66^ In the next step, the combination of these two approaches for creating permeable, mechanically strong, surface-textured hybrid structures could be promising in would healing applications.^67^ These novel insights have broad implications for the processing design of protein-based biomaterials. Apart from the impact on improved cell adhesion and proliferation studies, e.g., in skin tissue engineering, we envisage the possibility of using the structuring procedures for the preparation of environmentally friendly materials enabling passive radiative cooling systems.^68−70^

## Supporting Information Available

Additional experimental details, FTIR and roughness analysis, and cell proliferation (PDF)

## Abbreviations

SF: silk fibroin
RGD: arginylglycylaspartic acid
PDMS: polydimethyl sulfoxide
BG: breath figures
HFIP: hexafluoroisopropanol
DMSO: dimethyl sulfoxide
FTIR: Fourier transform infrared
ATR: attenuated total reflection
FSD: Fourier self-deconvolution, FID, fluorescein isothiocyanate-dextran
