# Supplementary material for: Silk Fibroin Surface Engineering Using Phase Separation Approaches for Enhanced Cell Adhesion and Proliferation
Source: ACS Appl Mater Interfaces. 2025 Feb 19;17(9):13702–12. doi: 10.1021/acsami.5c00874 (PMC11891832; doi:10.1021/acsami.5c00874)
Supplement: Supplementary file 1 — am5c00874_si_001.pdf [file am5c00874_si_001.pdf]

Supporting Information

Silk Fibroin Surface Engineering Using Phase Separation Approaches for Enhanced Cell Adhesion and Proliferation

Karolína Kocourková, Markéta Kadlečková, Erik Wrzecionko, Filip Mikulka, Eliška Knechtová, Petronela Černá, Martin Humenik and Antonín Minařík**

Table of Content

Supporting figures 3

Figure S1. *Optical microscopy images of different fibroin surfaces prepared by sequence dosing method at rotation – influence of water in the surface modifying solvent mixture* 3

Figure S2. *AFM measurements of film topography* 4

Figure S3. *Deconvolution of amide I bands* 5

Figure S4. *Detailed FTIR spectra of Amide I and Amide II band with effect of methanol vapor exposure time* 6

Figure S5. *Changes in surface topography in I. of Nano/Micro structured films* 7

Figure S6. *AFM images reflecting changes in surface topography of the films prepared using the rotational modification* 8

Figure S7. *Optical microscopy images of different fibroin surfaces prepared by sequence dosing method at rotation – influence of DMSO in the surface modifying solvent mixture* 9

Figure S8. *Optical microscopy images of different fibroin surfaces prepared by sequence dosing method at rotation – influence of process parameters on the macro topography formation* 10

Figure S9. *Optical profilometry images of different fibroin surfaces prepared by sequence dosing method at rotation – influence of the initial Silk II state of the SF films on the macro topography* 11

Figure S10. *A) Schematic representation of the process to prepare selectively labeled SF films by the sequenced dosing of solvent mixture enriched by fluorescein. B) Optical profilometry of the modified SF film. C) Fluorescence microscopy of the film in B at different magnification.* 12

Figure S11. *Distribution of pit diameters on the textured surface ROT 2 processed based on the analysis of 5 samples.* 13

Figure S12. *AFM images reflecting influence of the DMSO amount on the SF film texturing upon addition of one dose* 14

Figure S13. *AFM images reflecting changes in surface topography of the films prepared using the static modification* 15

Figure S14. *Apparatus scheme used in the fibroin surface modification with DMSO vapors via breath figures approach* 16

Figure S15. *Assessing cell proliferation of keratinocytes* 17

Figure S16. *Demonstration of cell nuclei localization for quantification of the cell number in A) – C) and cell shape definition for aspect ratio calculation in D) – F) as elaborated from fluorescence microscope images using ImageJ algorithms.* 17

Figure S17. *HaCaT proliferation on the SF films.* 18

Figure S18. *Schematics illustrating the processes of microextrusion printing (A) and EHD printing (B)*..... 19

Figure S19. *EHD printed fibroin grid in SILK I state (w/o posttreatment) A) before texturization by solvent mixture HFIP/H_2O /DMSO, B) after texturization; EHD printed fibroin grid in SILK II state (methanol posttreatment) C) before texturization, D) after texturization; E) and F) surface topography of C) and D) in detail obtained by AFM.* 20

Figure S20. *3D-printed fibroin grid obtained in the microextrusion process.*... 21

Figure S21. *Surface texturization of microextrusion 3D-printed fibroin structures visualized using A)–C) SEM and D)–F) AFM; A), D) smooth surface of the printed filament; B), E) surface of the printed filament after texturization by dispensing a solvent mixture of HFIP/H_2O /DMSO in a 7:4:0.2 ratio under rotation; C), F) surface of the printed filament after deposition of the solvent mixture onto a static substrate. Due to macroscopic thickness of the printed grids, no posttreatment was necessary to stabilize the structure against modification mixtures and to obtain textures.* 22

Supporting tables **23**

Table S1. *Protein secondary structure content from FSD in Figure S3A-F.* 23

Table S2. *Roughness parameters for the data in Figure S2 – silk surface topography in different form of protein secondary structure.* 23

Table S3. *Roughness parameters for the data in Figure 2 - different types of textured SF surfaces prepared using a sequenced dosing of solvent mixtures at substrate rotation.*... 23

Table S4. *Roughness parameters for the data in Figure S5 (ROT 1 image area 20×20 μm^2; ROT 2 image area 1×1 mm) – Changes in surface roughness after incubation in methanol and water* 24

Table S5. *Roughness parameters for the data in Figure S6 and S13 – Changes in surface roughness after incubation in cell culture medium*... 24

Table S6. *Roughness parameters for the data in Figure S9 (surface area 1×1 mm^2)– SF films with ROT 2 topography prepared after different times of methanol treatement*.. 25

Table S7. *Roughness parameters for the data in Figure 3 - different types of textured SF surfaces prepared on static substrate.*.. 25

Table S8. *Roughness parameters for the data in Figure S12 - influence of the DMSO amount on the SF film texturing upon addition of one dose of the modification mixture.*.. 26

Supporting Methods.. **27**

Supporting figures

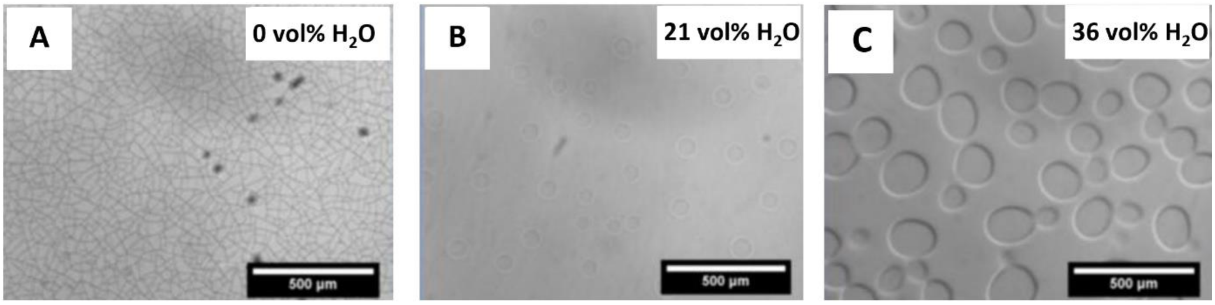

Figure S1. Optical microscopy images of different fibroin surfaces prepared by sequence dosing method at rotation – influence of water in the surface modifying solvent mixture: A) 0 %, B) 21 %, C) 36 % (v/v).

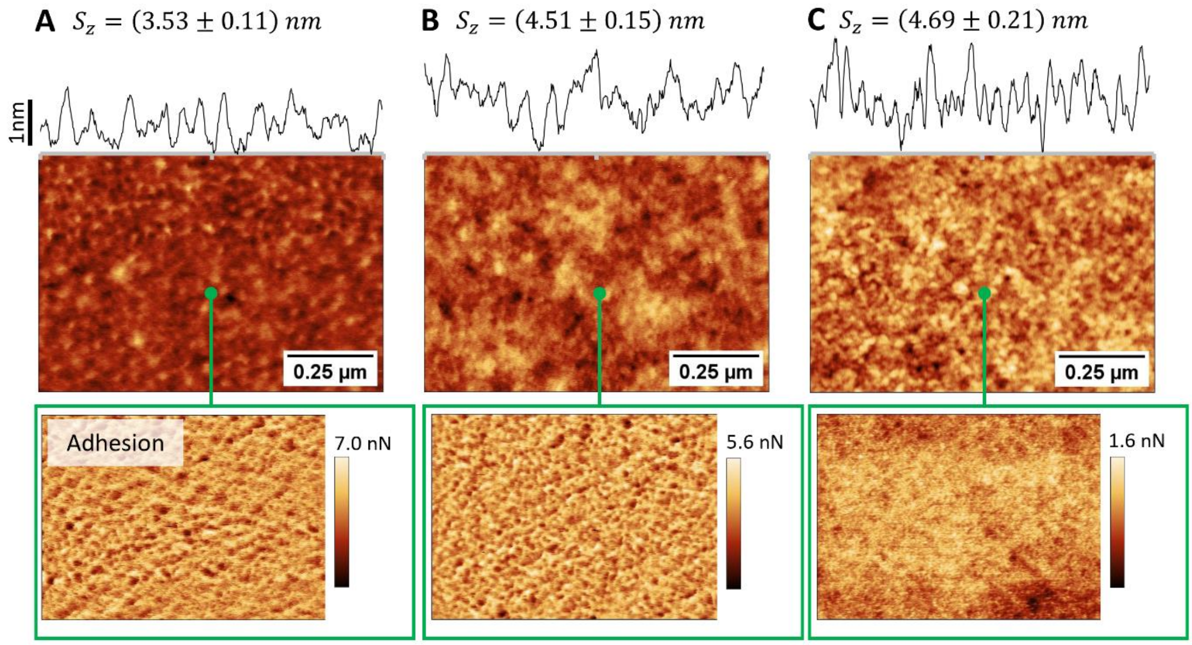

Figure S2. AFM measurements of film topography (upper panel) and adhesion (lower panel) after treatment: A) film in Silk I state after drying in N_2 atmosphere, B) film treated with 60% RH in transient Silk I/II state, C) film treated with methanol vapor in Silk II state,

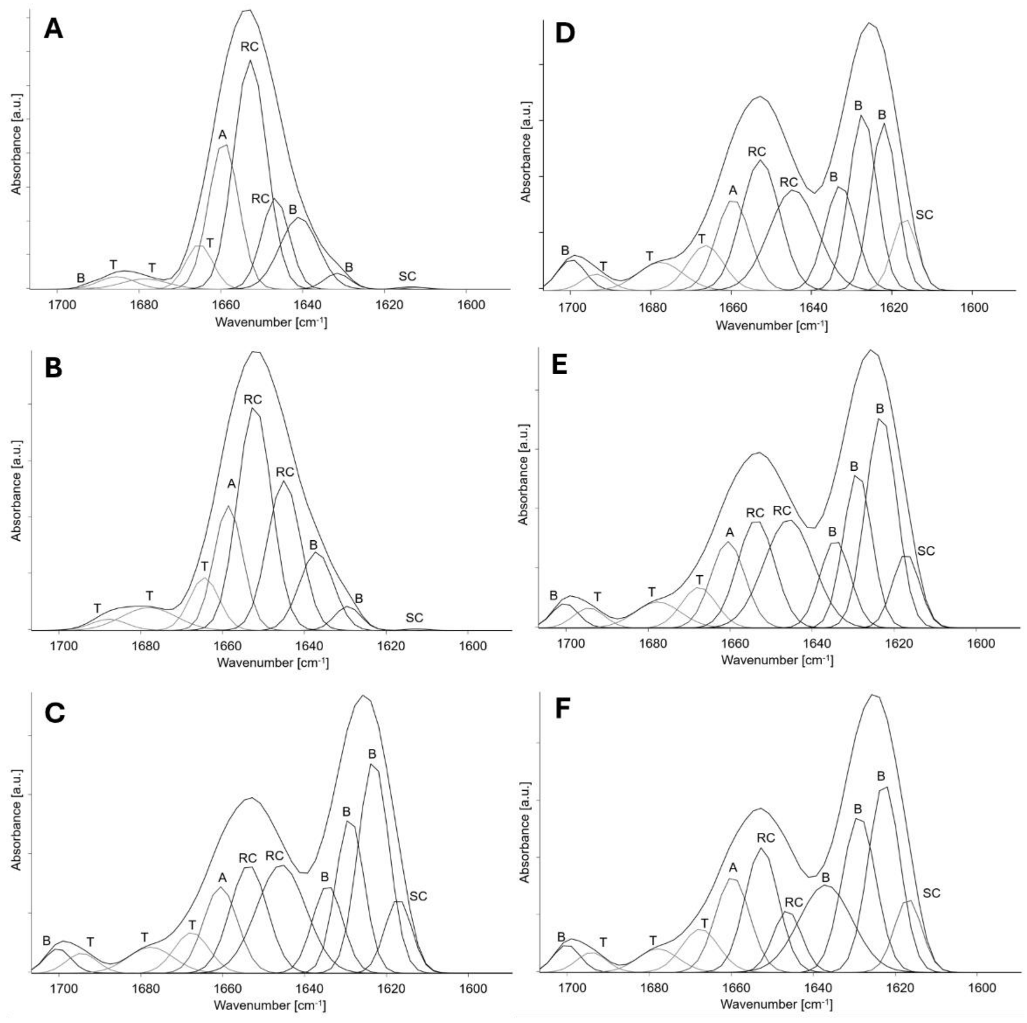

Figure S3. Deconvolution of amide I bands into individual subcomponents involving Gaussian curve fitting of A) Silk I, B) Silk I-II after 60% RH treatment, C) Silk II after methanol vapor treatment. Comparison of different methanol vapor exposure time for D) 24 h, E) 48 h, F) 72 h. Assigned secondary structural elements: SC: side chains, B: β-sheets, RC: random coil, A: α-helices, T: turns.

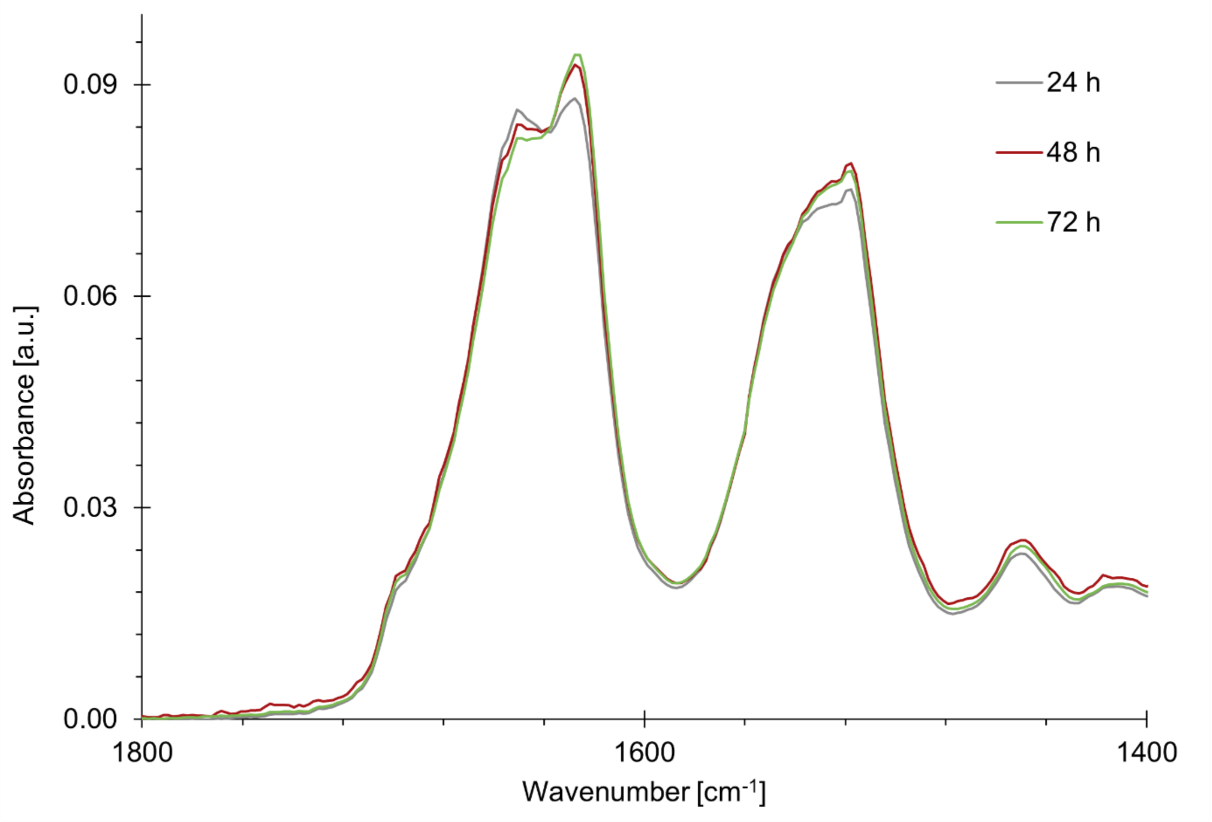

Figure S4. Detailed FTIR spectra of Amide I and Amide II band with effect of methanol vapor exposure time A) 24, B) 48, C) 72 h on SF conformation.

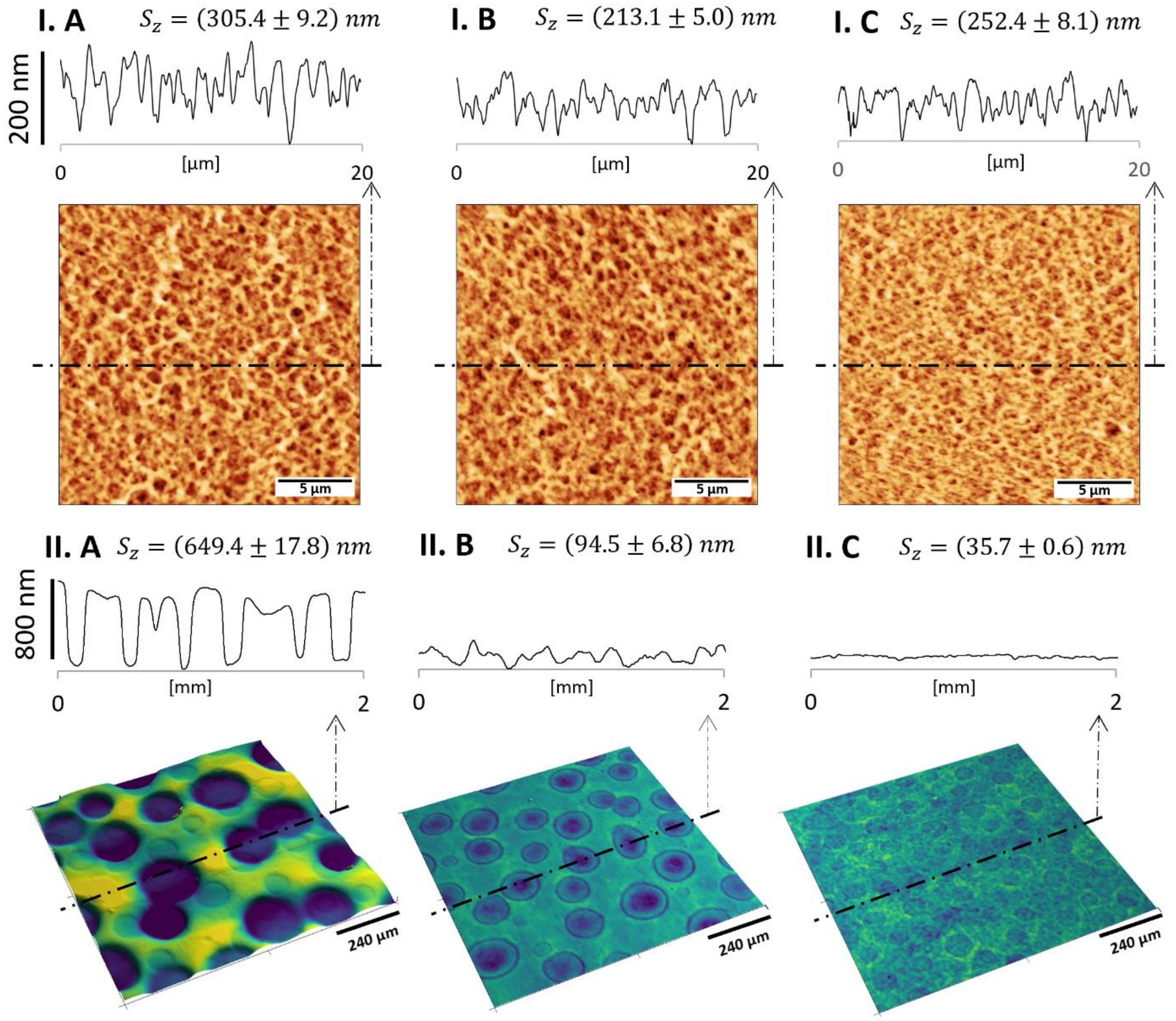

Figure S5. Changes in surface topography in I. of Nano/Micro structured films (Figure 2B, ROT 1), and in II. of Macro textures (Figure 2C, ROT 2); A) initial condition, B) after coverage with methanol for 24 h, C) after coverage with water for 24 h.

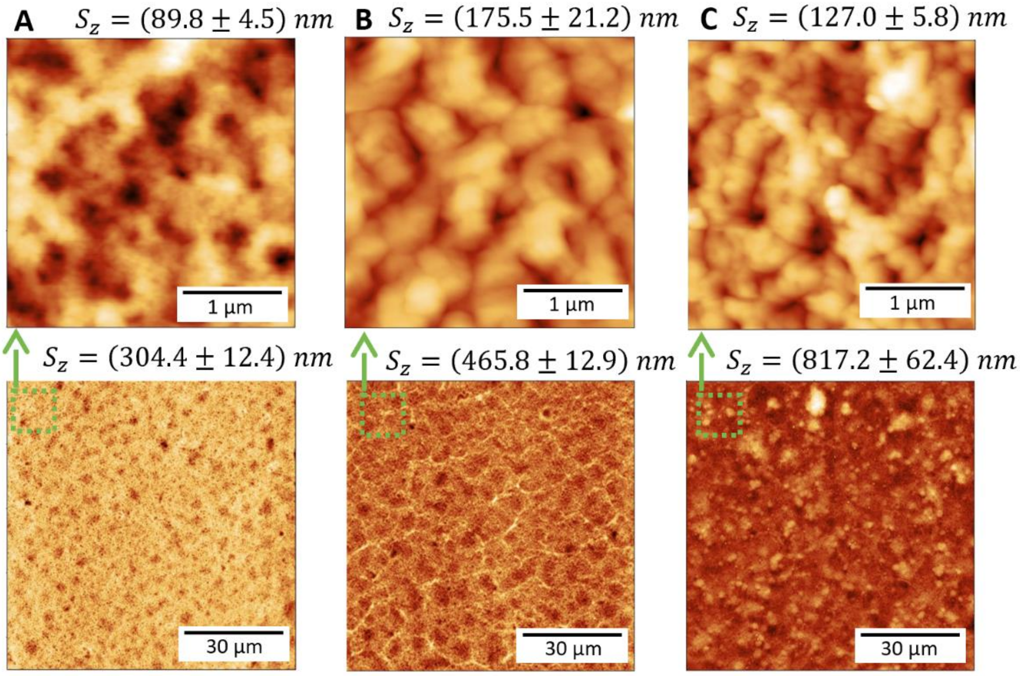

Figure S6. AFM images reflecting changes in surface topography of the films prepared using the rotational modification (Figure 2B) after incubation in cell culture medium at 37 °C for A) 0 days, B) 7 days, C) 14 days.

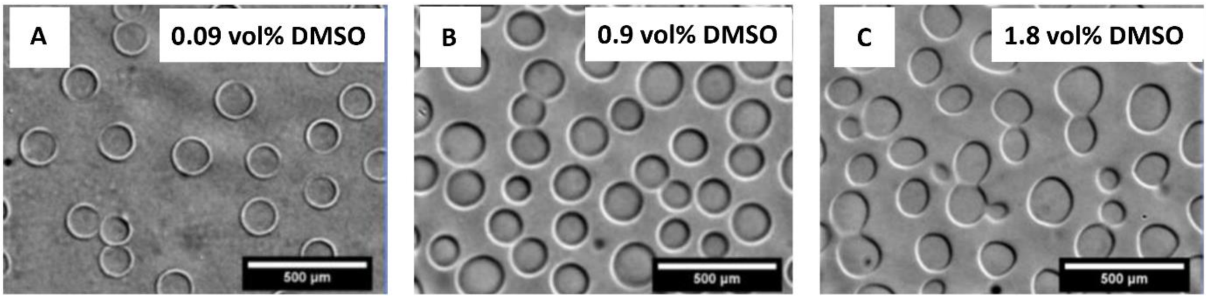

Figure S7. Optical microscopy images of different fibroin surfaces prepared by sequence dosing method at rotation – influence of DMSO in the surface modifying solvent mixture: A) 0.09 %, B) 0.9 %, C) 1.8 % (v/v).

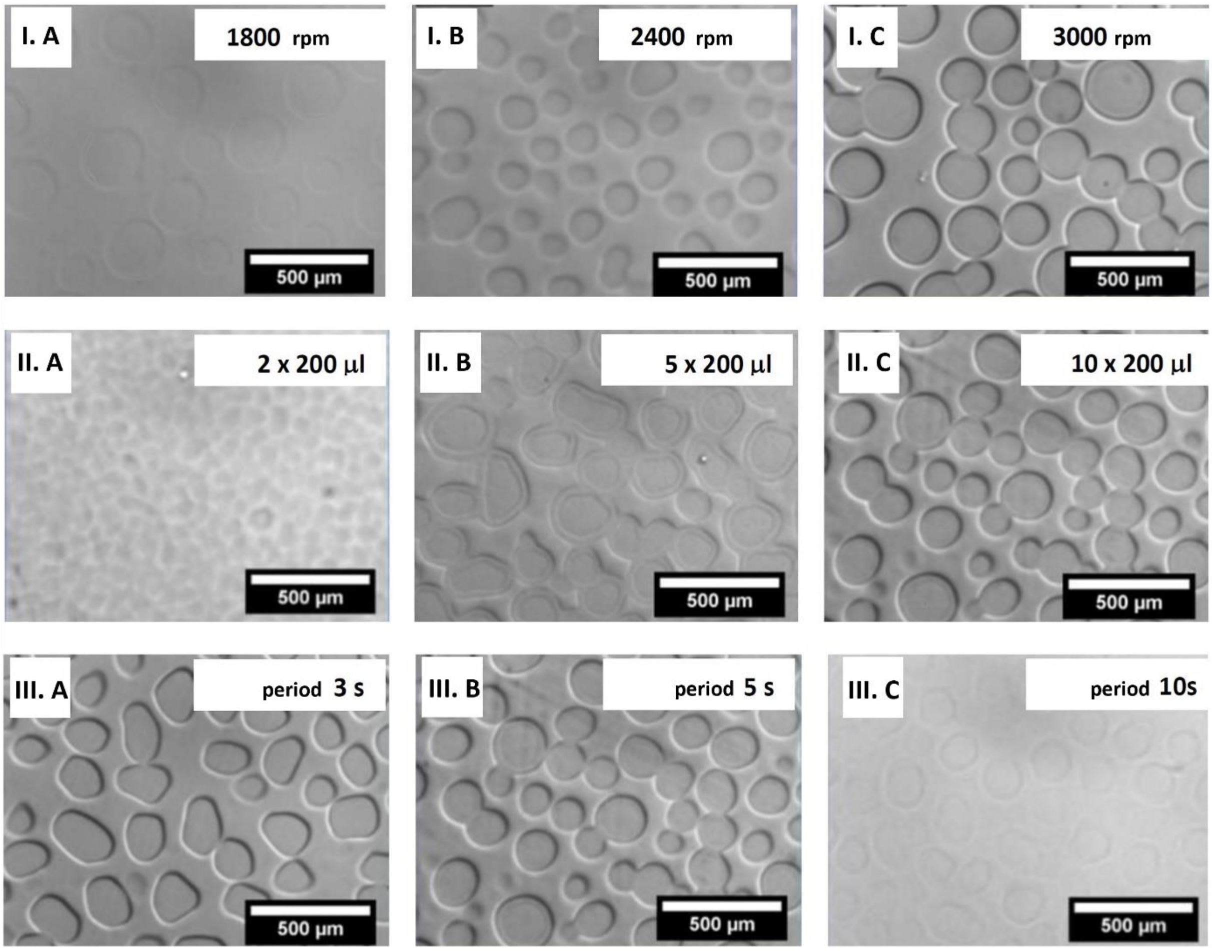

Figure S8. Optical microscopy images of different fibroin surfaces prepared by sequence dosing method at rotation – influence of process parameters on the macro topography formation: I. speed: A) 1800 rpm, B) 2400 rpm, C) 3000 rpm; II. number of doses: A) 2×200 µl, B) 5×200 µl, C) 10×200 µl, III. delay between the doses: A) 3 s, B) 5 s, C) 10 s.

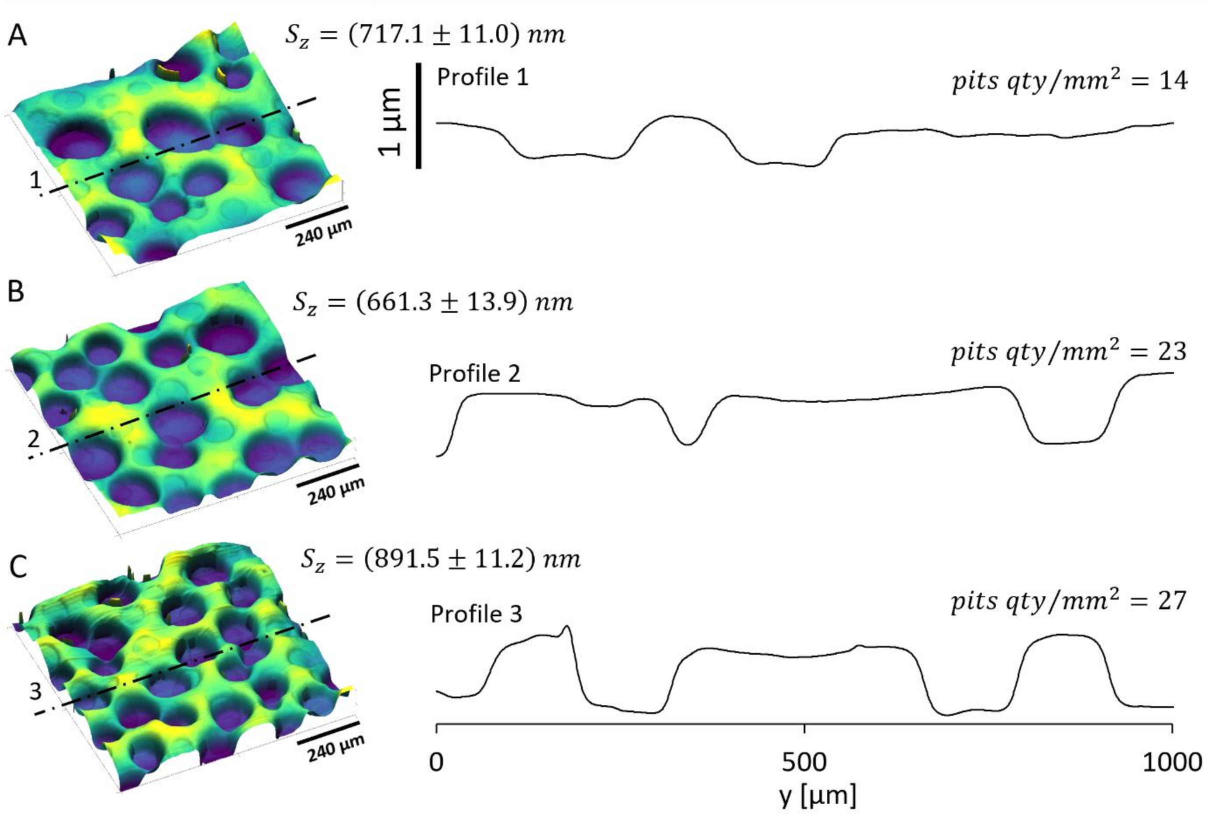

Figure S9. Optical profilometry images of different fibroin surfaces prepared by sequence dosing method at rotation – influence of the initial Silk II state of the SF films on the macro topography. Silk II films in Silk I states were treated with methanol for A) 24, B) 48, C) 72 h (Figure S1) before addition of the solvent mixture HFIP/H_2O/DMSO in ratio 7:4:0.2 using 10×200 µl doses, 2400 rpm substrate rotation and delay between the doses 5 s.

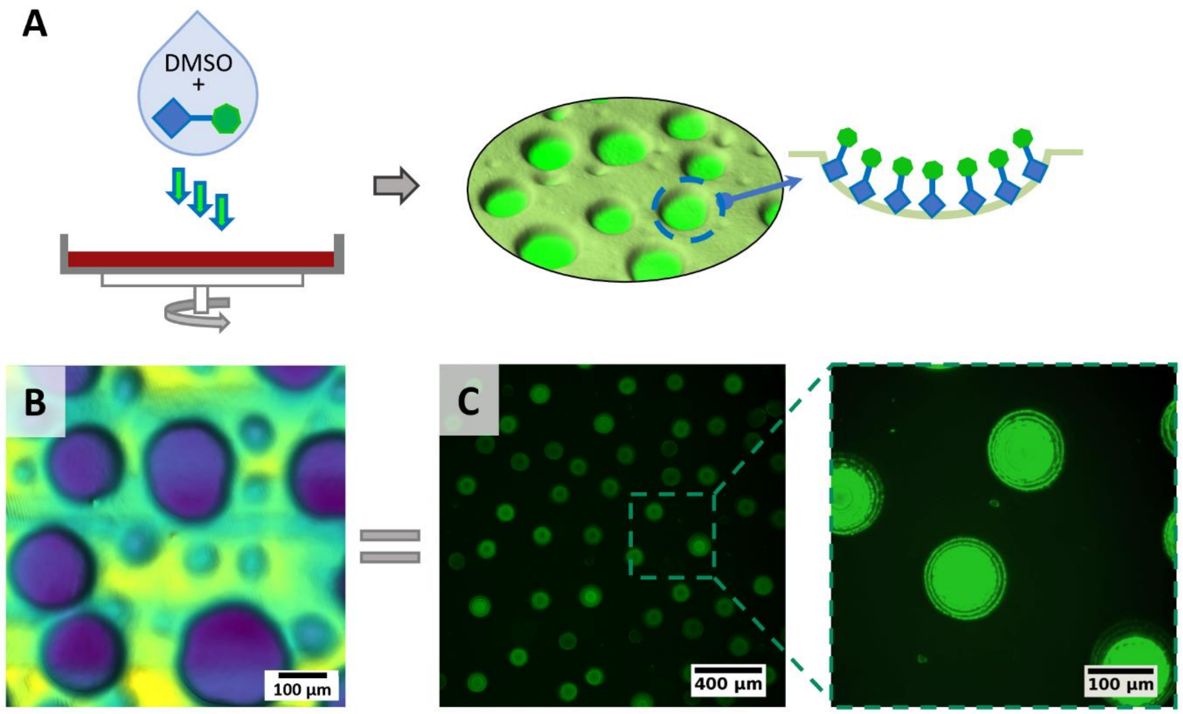

Figure S10. A) Schematic representation of the process to prepare selectively labeled SF films by the sequenced dosing of solvent mixture enriched by fluorescein. B) Optical profilometry of the modified SF film. C) Fluorescence microscopy of the film in B at different magnification.

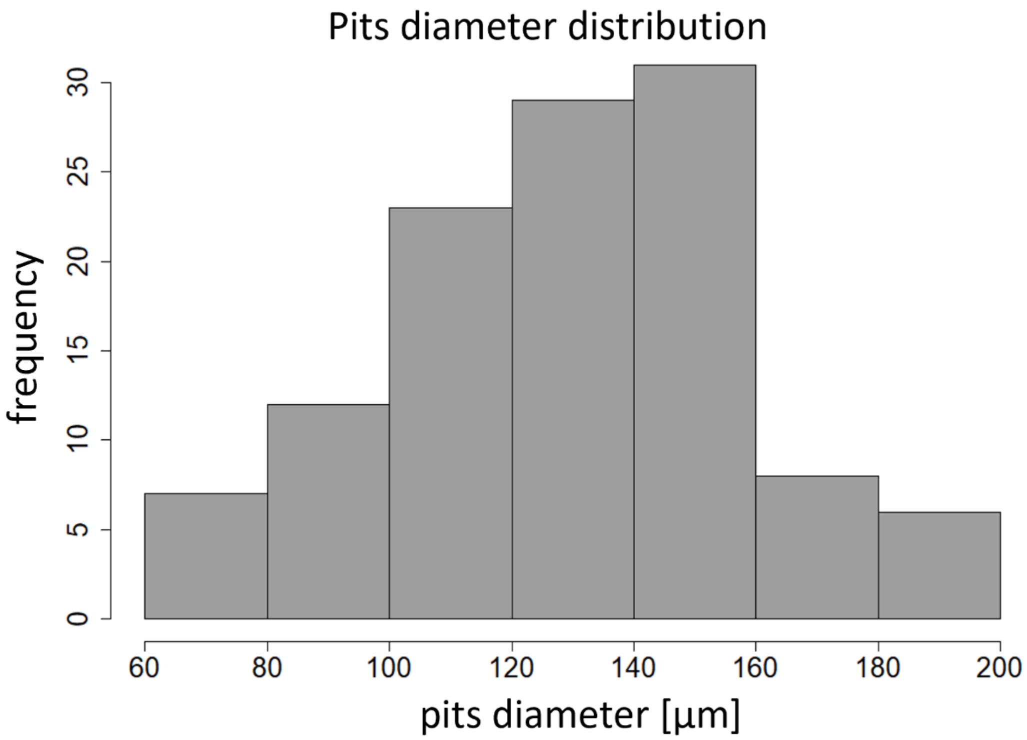

Figure S11. Distribution of pit diameters on the textured surface ROT 2 processed based on the analysis of 5 samples.

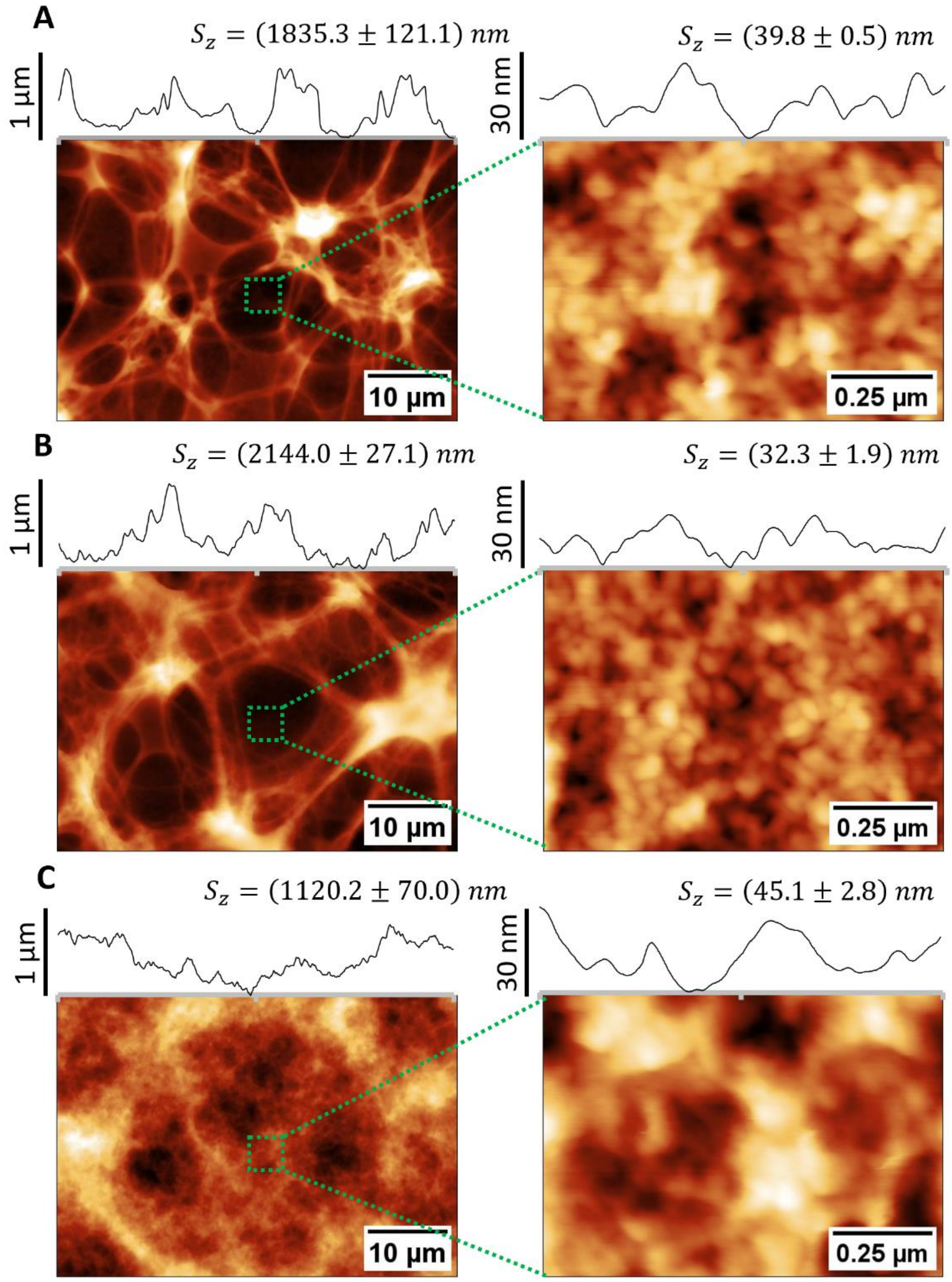

Figure S12. AFM images reflecting influence of the DMSO amount on the SF film texturing upon addition of one dose of the modification mixture composed HFIP/H_2O/DMSO (7:4:x ratio) and x DMSO in A) 0.9 %, B) 1.8 %, C) 3.6 % (v/v) on a non-rotating substrate.

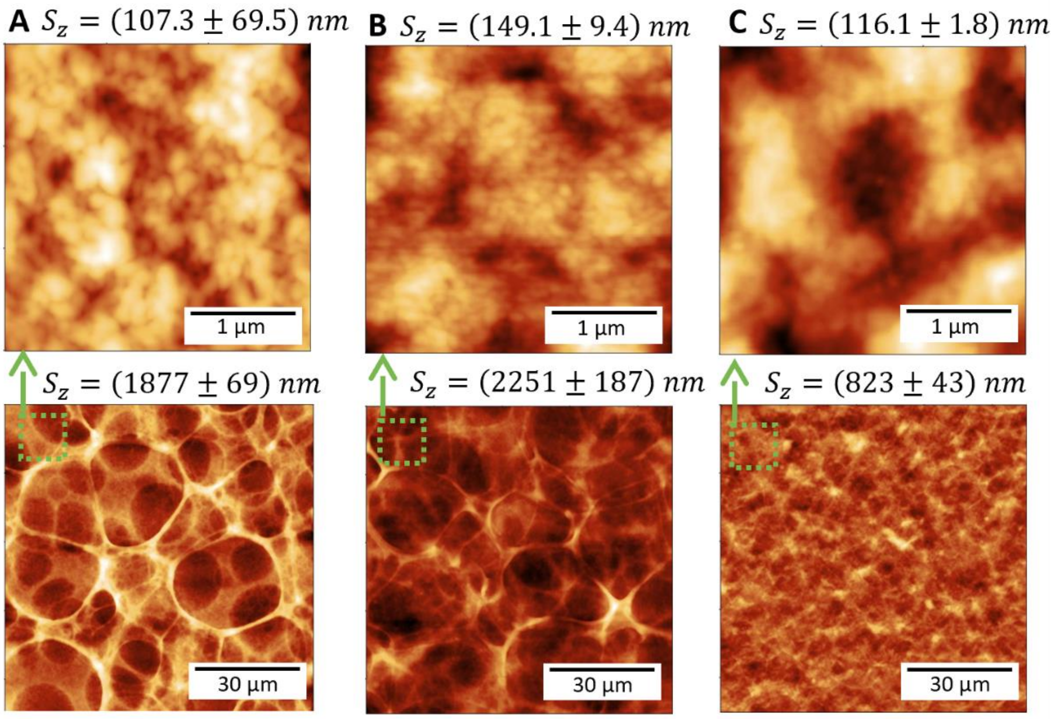

Figure S13. AFM images reflecting changes in surface topography of the films prepared using the static modification (Figure 3A) after incubation in cell culture medium at 37 °C for A) 0 days, B) 7 days, C) 14 days.

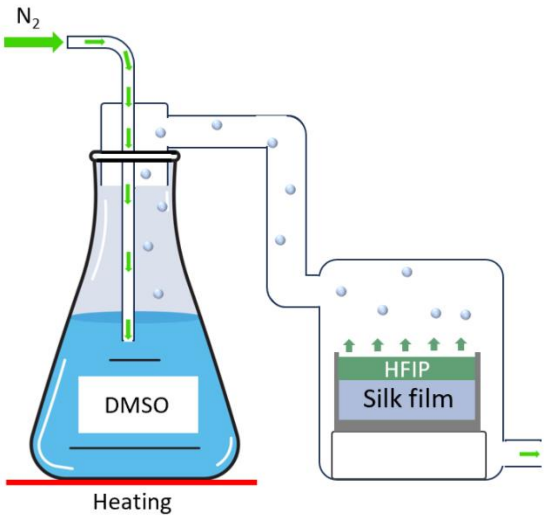

Figure S14. Apparatus scheme used in the fibroin surface modification with DMSO vapors via breath figures approach. Evaporation of the volatile HFIP is causing a cooling the silk film surface, which in turn leads to condensation of DMSO vapors transported through the chamber by an inert nitrogen atmosphere.

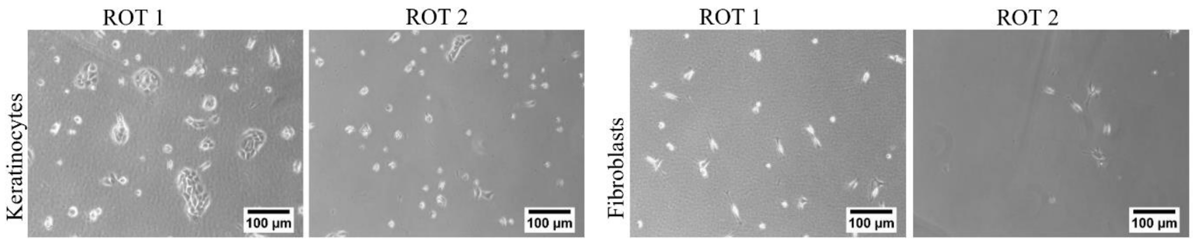

Figure S15. Assessing cell proliferation of keratinocytes (HaCaT) and fibroblasts (BALB/3T3), using an optical microscope on rotationally prepared substrates ROT 1 and ROT 2 after four days of culture.

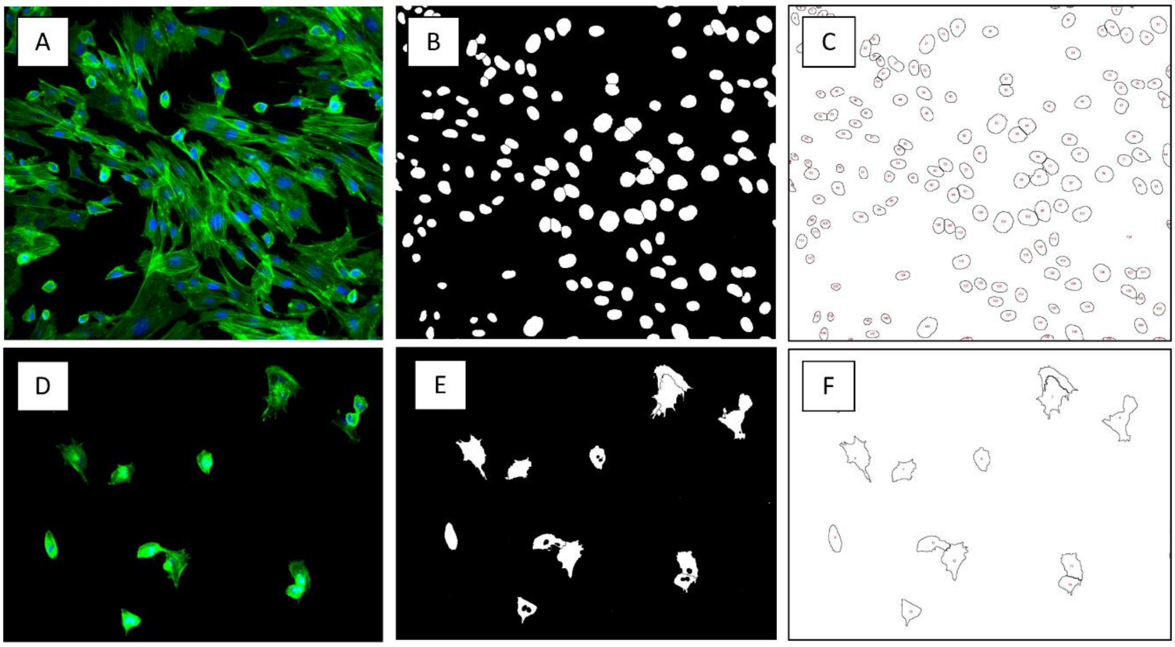

Figure S16. Demonstration of cell nuclei localization for quantification of the cell number in A) – C) and cell shape definition for aspect ratio calculation in D) – F) as elaborated from fluorescence microscope images using ImageJ algorithms.

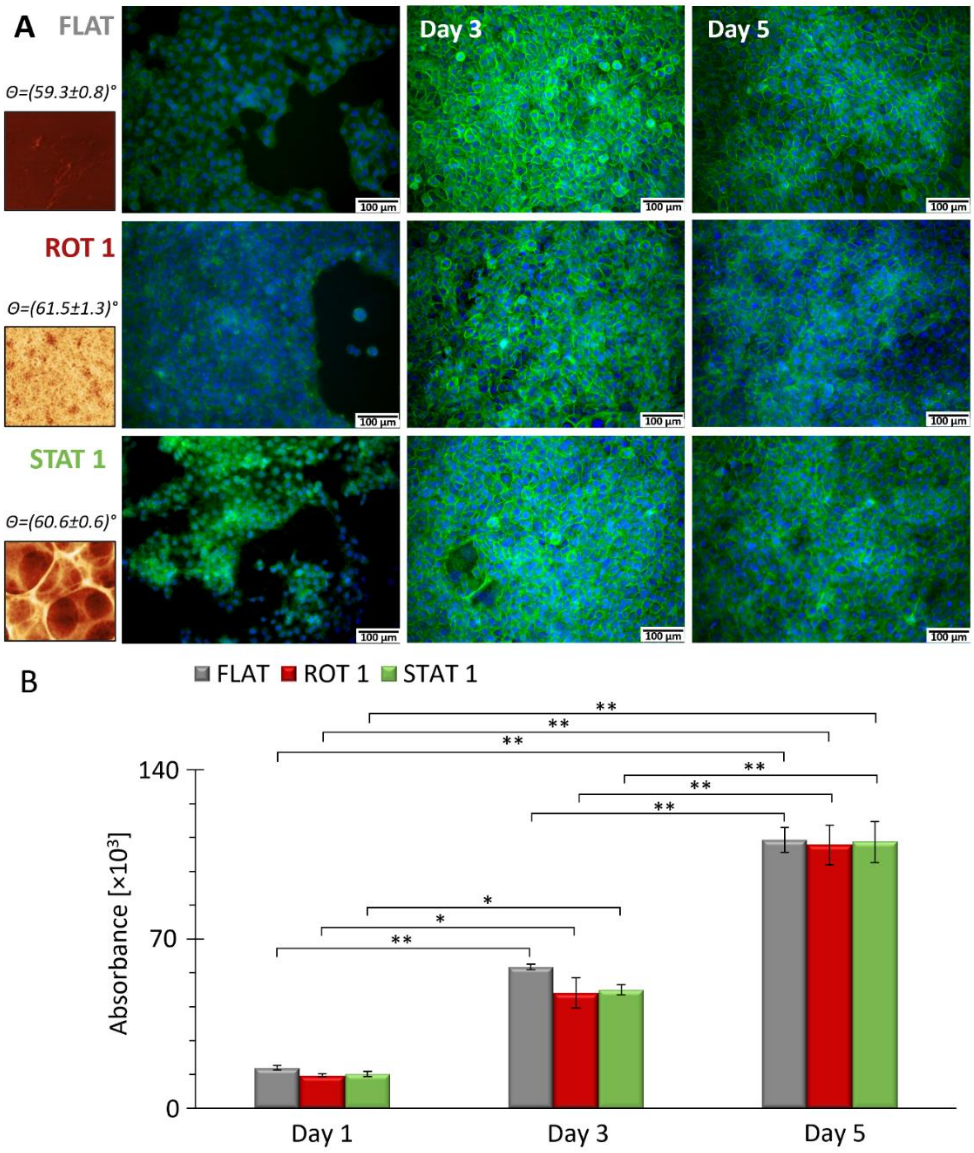

Figure S17. HaCaT proliferation on the SF films. A) the cells were incubated on different films indicated as FLAT = smooth SF surface, ROT 1 = surface with nano-micro pit-pattern prepared by sequenced solvent mixture dosing at substrate rotation, STAT 1 = surface prepared using static substrate and one dose of the modification mixture. The cells were monitored using fluorescence microscopy after nuclei staining (DAPI (blue) and actine staining (Phalloidin (green)) at the day 1, 3 and 5. B) Corresponding proliferation assays on the smooth and textured films. Statistically significant differences were determined using one-way ANOVA followed by a post-hoc Tukey test; * $p < 0.05$, ** $p < 0.01$.

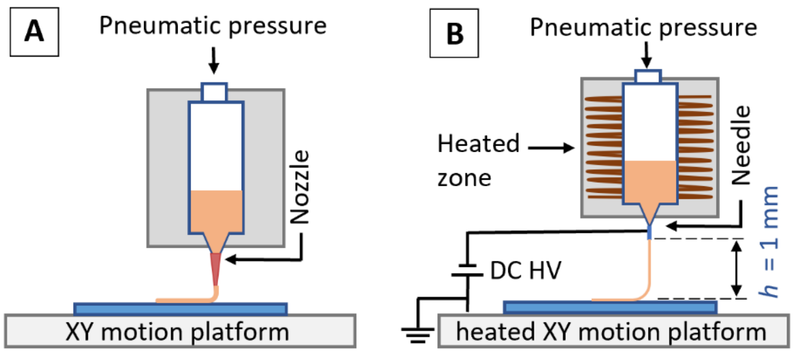

Figure S18. Schematics illustrating the processes of microextrusion printing (A) and EHD printing (B)

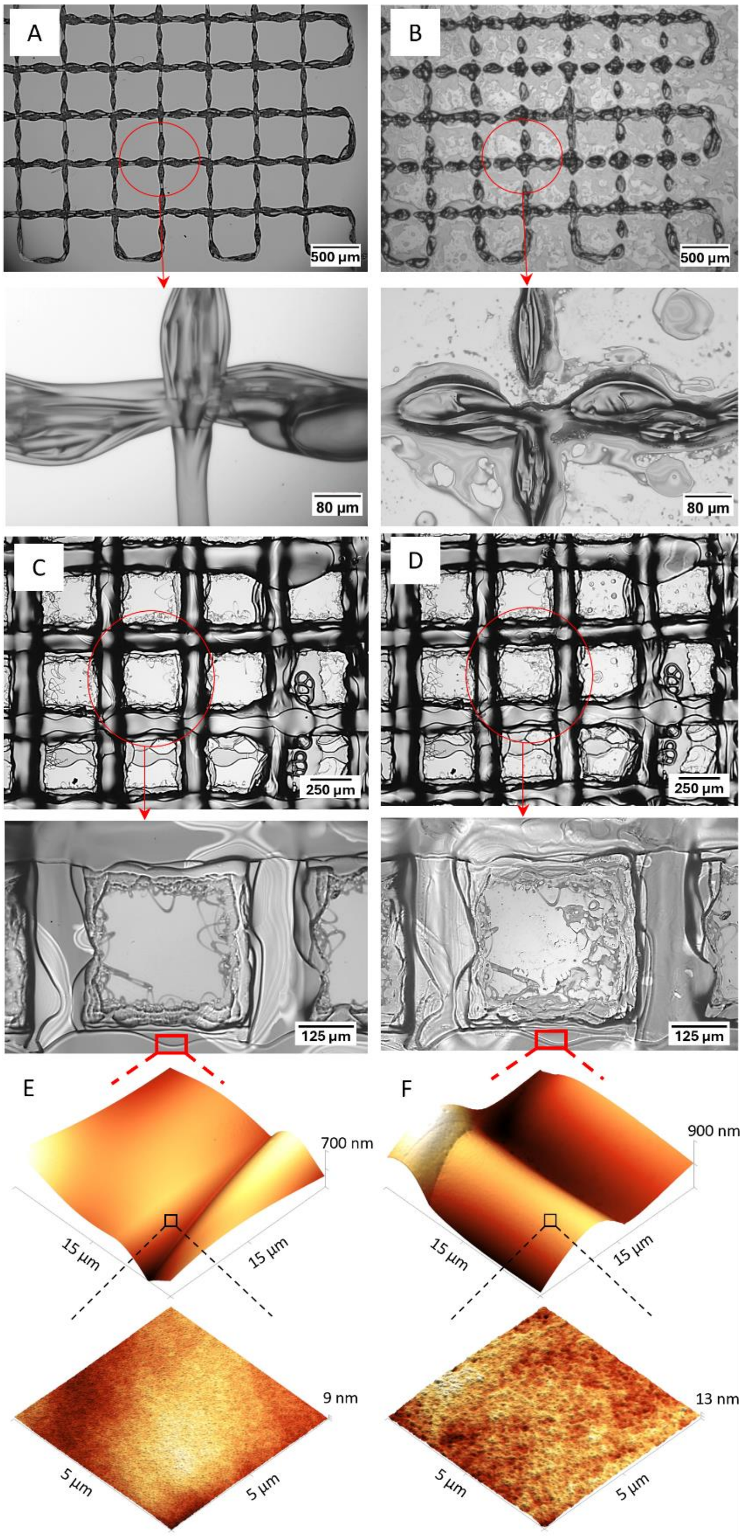

Figure S19. EHD printed fibroin grid in SILK I state (w/o posttreatment) A) before texturization by solvent mixture HFIP/H_2O/DMSO, B) after texturization; EHD printed fibroin grid in SILK II state (methanol posttreatment) C) before texturization, D) after texturization; E) and F) surface topography of C) and D) in detail obtained by AFM.

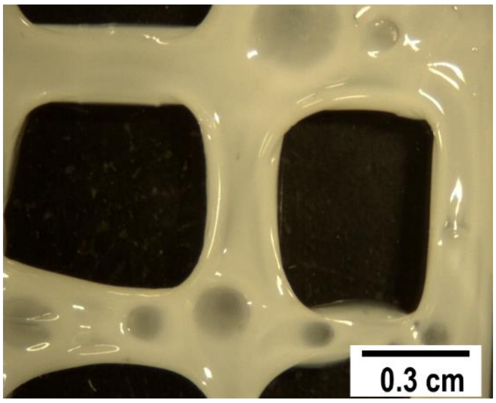

Figure S20. 3D-printed fibroin grid obtained in the microextrusion process.

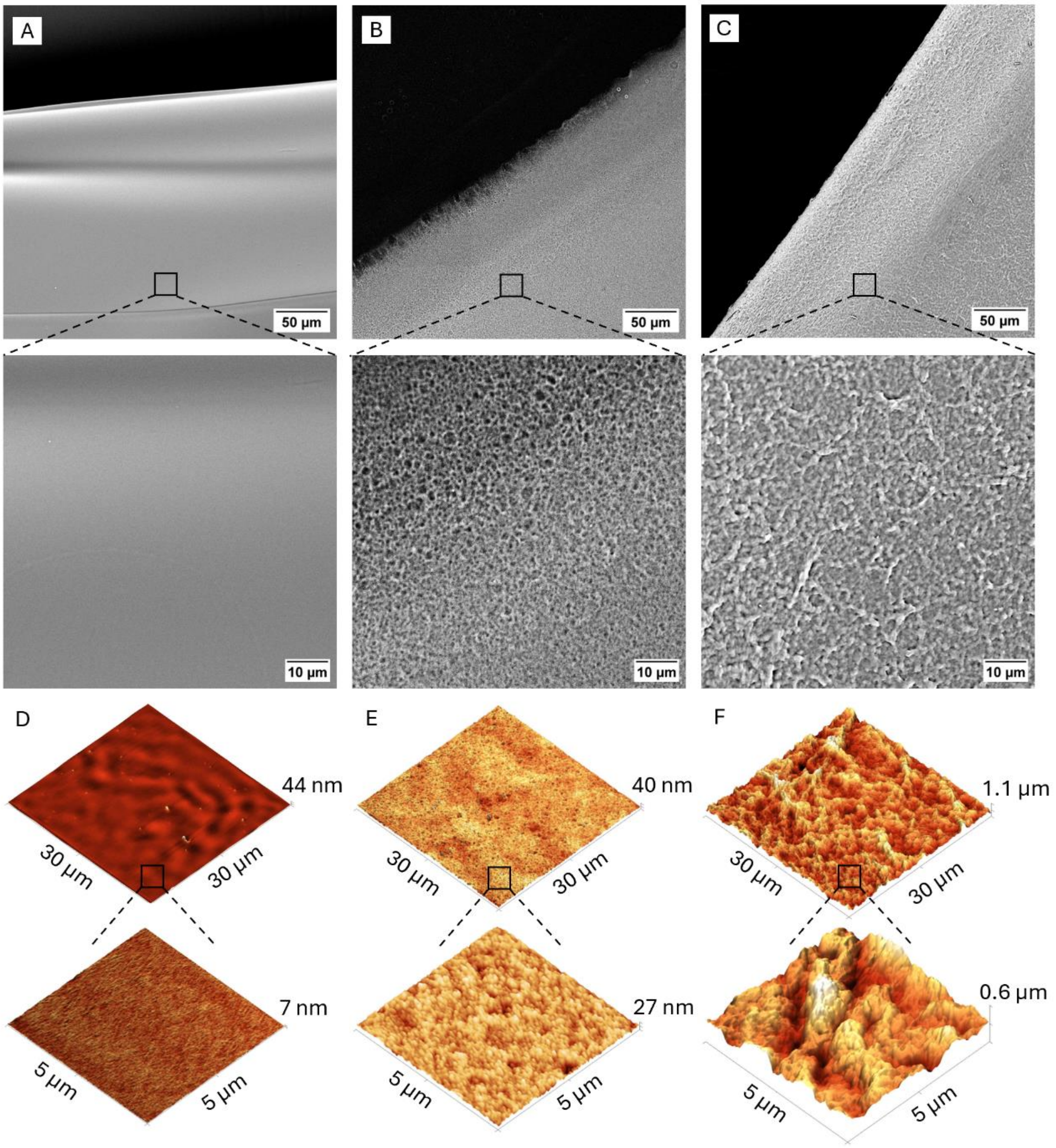

Figure S21. Surface texturization of microextrusion 3D-printed fibroin structures visualized using A)–C) SEM and D)–F) AFM; A), D) smooth surface of the printed filament; B), E) surface of the printed filament after texturization by dispensing a solvent mixture of HFIP/H_2O/DMSO in a 7:4:0.2 ratio under rotation; C), F) surface of the printed filament after deposition of the solvent mixture onto a static substrate. Due to macroscopic thickness of the printed grids, no posttreatment was necessary to stabilize the structure against modification mixtures and to obtain textures.

Supporting tables

Table S1. Protein secondary structure content from FSD in Figure S3A-F.

		Secondary structure content [%]				
	Wavenumber range [cm^{-1}]	Silk I N_2	Silk I-II RH 60	Silk II 24 h meOH	Silk II 48 h meOH	Silk II 72 h meOH
Side chains	1595-1615	0.3 ± 0.1	0.1 ± 0.0	6.7 ± 1.7	6.8 ± 1.4	8.1 ± 2.3
β-sheet	1616-1637,1697-1703	15.6 ± 0.5	15.8 ± 1.8	46.5 ± 4.7	50.1 ± 5.2	49.0 ±.5.5
random coil	1638-1655	50.7 ± 2.6	53.7 ± 1.6	26.4 ± 4.4	22.6 ± 5.9	23.1 ± 5.0
α-helices	1656-1662	22.2 ± 0.4	15.9 ± 0.7	9.8 ± 0.4	10.5 ± 1.2	9.8 ± 1.3
turns	1663-1696	11.2 ± 2.2	14.6 ± 0.4	10.7 ± 0.7	10.0 ± 0.2	10.0 ± 0.5

Table S2. Roughness parameters for the data in Figure S2 – silk surface topography in different form of protein secondary structure.

image area 1×1 μm^2	Sq [nm]	Sa [nm]	Sz [nm]
A) SILK I	0.39 ± 0.01	0.31 ± 0.01	3.53
B) SILK I-II	0.56 ± 0.01	0.45 ± 0.01	4.51 ± 0.15
C) SILK II	0.61 ± 0.02	0.48 ± 0.02	4.69 ± 0.21

Table S3. Roughness parameters for the data in Figure 2 - different types of textured SF surfaces prepared using a sequenced dosing of solvent mixtures at substrate rotation.

image area 0.7x0.7 mm^2	Sq [nm]	Sa [nm]	Sz [nm]
A) Flat	2.5 ±0.2	1.9 ± 0.2	24.2 ± 0.2
B) ROT 1	436.8 ± 17.1	354.6 ± 13.7	2473.3 ± 26.8
C) ROT 2	224.5 ± 14.7	196.4 ± 15.0	787.6 ± 31.7
D) ROT 1 + ROT 2	473.9 ± 25.8	387.6 ± 25.1	2502.8 ± 79.1
image area 20x20 μm^2	Sq [nm]	Sa [nm]	Sz [nm]
A) Flat	1.1 ± 0.1	0.7 ± 0.1	18.8 ± 1.3
B) ROT 1	39.6 ± 2.6	33.6 ± 0.2	304.4 ± 10.1

C) ROT 2	2.1 ± 0.1	1.5 ± 0.1	45.7 ± 2.7
D) ROT 1 + ROT 2	32.2 ± 0.4	25.5 ± 0.4	243.3 ± 6.7
image area 1×1 µm^2	Sq [nm]	Sa [nm]	Sz [nm]
A) Flat	0.41 ± 0.02	0.32 ± 0.02	3.44 ± 0.19
B) ROT 1	3.48 ± 0.13	2.76 ± 0.10	22.61 ± 1.07
C) ROT 2	1.03 ± 0.01	0.81 ± 0.01	8.76 ± 0.26
D) ROT 1 + ROT 2	5.07 ± 0.13	4.01 ± 0.12	35.59 ± 1.53

Table S4. Roughness parameters for the data in Figure S5 (ROT 1 image area 20×20 µm^2; ROT 2 image area 1×1 mm) – Changes in surface roughness after incubation in methanol and water

reference	Sq [nm]	Sa [nm]	Sz [nm]
I. ROT 1	37.1 ± 2.9	33.7 ± 0.2	305.4 ± 9.2
II. ROT 2	195.5 ± 5.2	178.6 ± 5.5	649.4 ± 17.8
methanol 24 h	Sq [nm]	Sa [nm]	Sz [nm]
I. ROT 1	28.8 ± 0.3	23.0 ± 0.2	213.1 ± 5.0
II. ROT 2	13.9 ± 0.4	11.4 ± 0.3	94.5 ± 6.8
water 24 h	Sq [nm]	Sa [nm]	Sz [nm]
I. ROT 1	30.0 ± 0.4	23.9 ± 0.4	252.4 ± 8.1
II. ROT 2	4.2 ± 0.1	3.3 ± 0.1	35.7 ± 0.6

Table S5. Roughness parameters for the data in Figure S6 and S13 – Changes in surface roughness after incubation in cell culture medium

day 0	Sq [nm]	Sa [nm]	Sz [nm]
I. ROT 1			
90x90 µm^2	31.0 ± 0.7	24.3 ± 0.5	304.4 ± 12.4
3x3 µm^2	16.8 ± 1.2	13.7 ± 1.1	89.8 ± 4.5

II. STAT 1			
90x90 μm²	319.3 ± 5.1	260.0 ± 3.4	1877.3 ± 69.5
3x3 μm²	18.3 ± 1.6	14.8 ± 1.3	107.3 ± 7.0
day 7			
I. ROT 1			
90x90 μm²	47.3 ± 3.5	39.0 ± 1.2	465.8 ± 12.9
3x3 μm²	27.9 ± 1.4	22.4 ± 0.9	175.5 ± 21.2
II. STAT 1			
90x90 μm²	345.3 ± 23.1	272.5 ± 17.6	2251.0 ± 186.8
3x3 μm²	25.9 ± 0.5	20.9 ± 0.4	149.1 ± 9.4
day 14			
I. ROT 1			
90x90 μm²	93.7 ± 6.4	71.7 ± 3.6	817.2 ± 62.4
3x3 μm²	19.7 ± 1.6	15.4 ± 1.4	127.0 ± 5.8
II. STAT 1			
90x90 μm²	25.9 ± 0.8	21.6 ± 0.9	116.1 ± 1.8
3x3 μm²	104.4 ± 4.5	81.7 ± 3.8	823.3 ± 43.1

Table S6. Roughness parameters for the data in Figure S9 (surface area 1×1 mm²)– SF films with ROT 2 topography prepared after different times of methanol treatement

Methanol treatement	Sq [nm]	Sa [nm]	Sz [nm]
A) 24 h	192.2 ± 4.5	167.8 ± 4.7	717.1 ± 11.0
B) 48 h	197.9 ± 5.0	180.9 ± 5.3	661.3 ± 13.9
C) 72 h	257.3 ± 8.7	229.2 ± 8.7	891.5 ± 11.2

Table S7. Roughness parameters for the data in Figure 3 - different types of textured SF surfaces prepared on static substrate.

image area 0.7x0.7 mm²	Sq [nm]	Sa [nm]	Sz [nm]
A) STAT 1	336.7 ± 11.2	274.8 ± 9.4	2400.5 ± 50.4
B) STAT 2	14.3 ± 0.5	10.8 ± 0.5	200.0 ± 28.8
image area 50x50 μm²	Sq [nm]	Sa [nm]	Sz [nm]
A) STAT 1	316.5 ± 15.8	251.5 ± 13.9	1774.8 ± 47.3

B) STAT 2	22.0 ± 1.8	16.5 ± 1.7	181.4 ± 6.4
image area 8x8 µm^2	Sq [nm]	Sa [nm]	Sz [nm]
A) STAT 1	26.9 ± 0.4	21.6 ± 0.5	181.1 ± 3.7
B) STAT 2	16.3 ± 1.0	10.5 ± 0.8	121.2 ± 8.4

Table S8. Roughness parameters for the data in Figure S12 - influence of the DMSO amount on the SF film texturing upon addition of one dose of the modification mixture.

image area 50x50 µm^2			
DMSO amount (% v/v)	Sq [nm]	Sa [nm]	Sz [nm]
A) 0.9	270.3 ± 14.1	205.5 ± 10.6	1835.3 ± 121.1
B) 1.8	449.5 ± 29.1	350.8 ± 22.5	1619.5 ± 539.6
C) 3.6	193.5 ± 20.1	155.9 ± 16.4	1120.2 ± 70.0
image area 1x1 µm^2			
DMSO amount (% v/v)	Sq [nm]	Sa [nm]	Sz [nm]
A) 0.9	6.9 ± 0.4	5.6 ± 0.4	39.8 ± 0.5
B) 1.8	5.2 ± 0.3	4.3 ± 0.2	32.3 ± 1.9
C) 3.6	9.0 ± 0.4	7.5 ± 0.4	45.1 ± 2.8

Supporting Methods

Selective surface labeling

Films in the Silk II state were modified using sequential dispensing of the solution onto a rotating substrate. The modification solution contained HFIP, water and DMSO in a 7:4:0.2 volume ratio, whereas DMSO part contained 0.1 % (w/v) of fluorescein isothiocyanate dextran. Films were modified using 10×200 µl of this mixture at a dosing period of 5 s and 2400 rpm.

Extrusion printing of silk fibroin and surface texturization

A 10 wt.% fibroin solution was processed into a 10×10×0.5 mm grid structure using a 3D printer, Cellink BioX (Gothenburg, Sweden), with the following specifications: a polypropylene conical nozzle with a diameter of 0.41 mm, a 3 mL polypropylene syringe, a microextrusion syringe pump printhead, and a PS Petri dish as the printbed. The printing speed ranged from 9 to 12 $mm \cdot s^{-1}$, and the applied pressure was 50–90 kPa. The process is schematically illustrated in Figure S18 A.

The printed structures were dried at room temperature (23 °C) with a relative humidity of 50–60% and subsequently textured using modified approaches for rotational and static surface modifications. The rotational modification was performed using a custom-built device, where a solvent mixture of HFIP/H_2O/DMSO in a 7:4:0.2 ratio was dispensed onto the rotating substrate. The process parameters included a rotation speed of 1500 rpm, a dosing volume of 100 µL, a dosing interval of 5 seconds, and 4 repeated doses.

The static modification was performed by dispensing 500 µL of the aforementioned solvent mixture into a dish containing the printed grid. The solution was left on the grid for one hour, after which the grid was dried using rotational drying.
